# *Hemerocallis citrina* Baroni (Daylily): A Comprehensive Review of Flavonoids and Phenolic Compounds—Extraction, Phytochemistry, Biological Activities, and Food Applications (2000–2026)

**DOI:** 10.3390/foods15203643

**Published:** 2026-10-14

**Authors:** Salman Khan, Xing Zhang, Muhammad Junaid, Shaohua Huang

**Affiliations:** 1 Institute of Drug Discovery and Technology, Ningbo University, Ningbo 315211, China; 2 Zhejiang-Pakistan Joint Laboratory on R&D of Herbal Medicines, Institute of Drug Discovery and Technology, Ningbo University, Ningbo 315211, China; 3 Health Science Center, Ningbo University, Ningbo 315211, China

**Keywords:** *Hemerocallis citrina* Baroni, daylily, flavonoids, phenolic compounds, phenolic acids, extraction, biological activities, functional food, traditional Chinese medicine

## Abstract

*Hemerocallis citrina* Baroni, commonly known as daylily or “Huang Hua Cai,” is a well-established traditional medicinal and edible plant widely cultivated in East Asia for more than two millennia. Its flower buds, stems, leaves, and roots are rich in flavonoids and phenolic compounds, which constitute the principal bioactive phytochemicals responsible for a wide range of pharmacological effects, including antioxidant, anti-inflammatory, neuroprotective, antidepressant, and sleep-improving activities. Although several studies have investigated the chemical composition of *Hemerocallis citrina* Baroni, a comprehensive and up-to-date review specifically focused on the flavonoids and phenolic compounds of this plant remains lacking. In this review, we systematically summarize the research progress from 2000 to 2026 on the extraction methods, structural identification, phytochemical profiles, and biological activities of flavonoids and phenolic compounds from *H. citrina* flower buds, stems, and leaves. Advanced analytical techniques, such as UPLC-PDA/MS, UHPLC-QQQ-MS/MS, and HPLC-Q-TOF-MS, have revealed a diverse array of these compounds, including quercetin, kaempferol, and isorhamnetin glycosides, as well as various caffeoyl- and *p*-coumaroylquinic acids. The health-promoting effects of these polyphenols, together with current knowledge on their mechanisms of action and potential applications in functional foods and traditional Chinese medicine, are critically discussed. By consolidating recent phytochemical and pharmacological findings, this review provides a scientific foundation for the further development and utilization of *H. citrina* flavonoids and phenolic compounds as valuable resources for functional foods, nutraceuticals, and natural therapeutics.

## 1. Introduction

The genus *Hemerocallis* (family Asphodelaceae, subfamily Hemerocallidoideae) comprises approximately 20 species native to central and northern China, the Korean Peninsula, Japan, and parts of Eastern Europe [1]. Among these, *Hemerocallis citrina* Baroni, commonly known as daylily, “Huang-Hua-Cai” (yellow flower vegetable), “Jin-Zhen-Cai” (golden needle vegetable), or “Wang-You-Cao” (forget-one’s-sadness plant), has occupied a unique dual role as both a dietary staple and a medicinal herb for more than 2000 years [2,3]. The dried flower buds are listed in the *Compendium of Materia Medica* for their sedative, mood-elevating, and lactation-promoting properties [3], and the 2021 edition of the Japanese Pharmacopoeia recognizes daylily as an essential natural medicine for treating insomnia and relieving depression [4]. In folk medicine, the plant has long been used to reduce swelling, relieve pain, stop bleeding, lower blood pressure, and treat abscesses [2,4].

*Hemerocallis citrina* Baroni is a traditional medicinal and edible plant widely cultivated in China. Its flower buds are particularly rich in flavonoids and polyphenols, which have been identified as the principal secondary metabolites responsible for a wide range of biological activities, including antioxidant, anti-inflammatory, antidepressant, and neuroprotective effects. Although numerous studies have investigated the chemical composition and pharmacological properties of daylily flowers, a comprehensive and up-to-date review specifically focused on the flavonoids and polyphenols of *H. citrina* flower buds is still lacking. Therefore, the present review systematically summarizes the research progress on the extraction, identification, structural characteristics, and biological activities of flavonoids and polyphenolic compounds from *H. citrina* flower buds, aiming to provide a scientific basis for their further development and utilization in functional foods and natural medicines [2,5,6,7,8].

**Figure 1:**
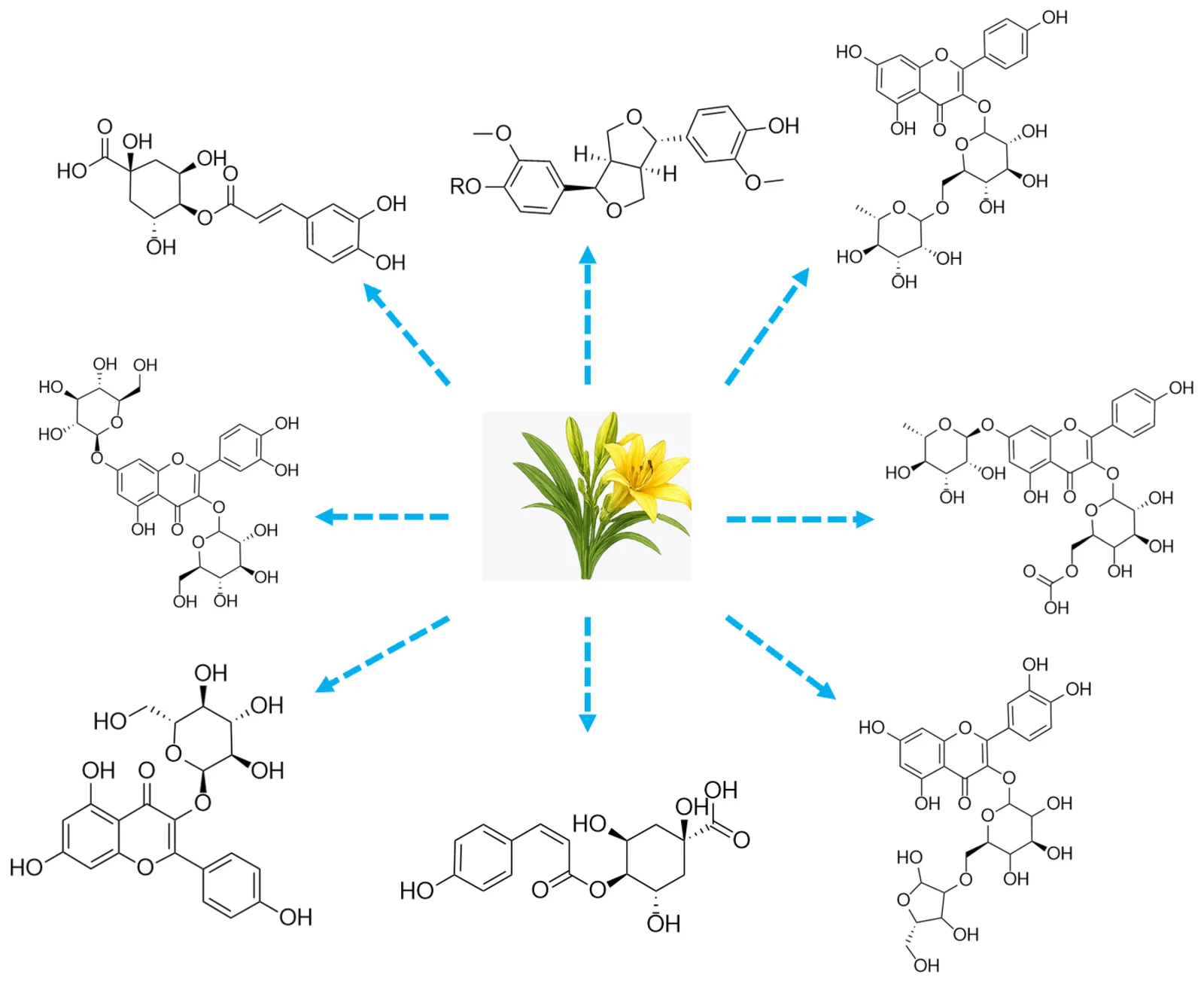
Flavonoids and Phenolics from *Hemerocallis citrina* Baroni.

Flavonoids and phenolic acids constitute the major low-molecular-weight bioactive constituents in the flower buds of *Hemerocallis citrina* (Figure 1). Recent phytochemical investigations using advanced techniques such as UPLC-PDA/MS, UHPLC-QQQ-MS/MS, and HPLC-Q-TOF-MS have revealed a rich diversity of these compounds, including quercetin, kaempferol, and isorhamnetin glycosides, as well as various caffeoyl- and *p*-coumaroylquinic acids [5,9,10,11,12]. These polyphenols have been shown to contribute significantly to the antioxidant, anti-inflammatory, antidepressant, and other health-promoting effects of daylily. However, the available literature encompasses a broad range of secondary metabolites, plant parts, analytical approaches, and biological models, and the reported findings are therefore dispersed across different aspects of *H. citrina* research [7,8,9,13]. Integrating these findings within a species-focused framework is important for clarifying the current phytochemical evidence, methodological approaches, and relationships between chemical composition, biological activity, safety, and potential food applications.

To provide a comprehensive species-level perspective, the present review adopts *H. citrina* Baroni as the focal species while maintaining flowers and flower buds as the principal plant materials of interest because of their importance as edible and traditionally utilized materials and their extensive investigation for flavonoids and phenolic compounds. Relevant evidence from leaves, stems, roots, and other plant tissues is considered selectively where it provides direct information on the occurrence, distribution, characterization, biosynthesis, biological properties, processing, safety, or authentication of the target phytochemicals. The review integrates evidence on the occurrence and characterization of flavonoids and phenolic compounds with their extraction and isolation, analytical identification, biological activities, nutritional and food-related applications, safety and toxicity, processing, and species authentication. Information concerning other chemical classes, including polysaccharides, anthraquinones, essential oils, and proteins, as well as genomic or biosynthetic characteristics, is included only when it provides relevant supporting context for understanding the phytochemistry, biological potential, safety, processing, authentication, or utilization of *H. citrina*. Such information is not intended to constitute independent major themes of the review. Evidence derived from other *Hemerocallis* species is clearly distinguished from species-specific evidence and is used only where appropriate for comparative or contextual purposes. Accordingly, the review provides an integrated, species-focused synthesis of the current knowledge of *H. citrina*, with flavonoids and phenolic/polyphenolic compounds remaining its central phytochemical focus.

## 2. Literature Search and Review Methodology

### 2.1. Review Design and Scope

This study was conducted as a comprehensive narrative review supported by a structured literature search and a documented study-selection process. The approach was designed to provide a transparent and reproducible basis for identifying and evaluating the available evidence concerning *Hemerocallis citrina* Baroni, with particular emphasis on flavonoids and phenolic/polyphenolic compounds. The review covered literature published from January 2000 to September 2026, with the final search conducted in September 2026 immediately before manuscript submission. The principal focus was on compounds reported from flower buds and flowers, which represent the major edible and traditionally utilized plant materials. Information on stems, leaves, roots, and other plant parts was considered selectively when directly relevant to the phytochemistry, extraction, biological activity, safety, authentication, processing, or utilization of *H. citrina*. Nutritional characteristics, extraction technologies, analytical approaches, biological activities, safety and toxicity, species authentication, and food applications were included as complementary aspects of the review. Because the available studies differed considerably in plant material, experimental design, analytical methodology, and biological models, the evidence was synthesized narratively rather than quantitatively, and no meta-analysis was performed. Following the screening and evaluation process, 99 references were selected for inclusion in the final review.

### 2.2. Literature Search Strategy

Literature searches were conducted using the Scopus and Web of Science Core Collection databases for publications from January 2000 to September 2026. The search strategy combined terms related to *H. citrina* with keywords describing its phytochemical composition, extraction, biological activities, safety, authentication, processing, and food applications. Principal search terms included “*Hemerocallis citrina*”, “*Hemerocallis citrina Baroni*”, “*daylily*”, “*flavonoids*”, “*phenolic compounds*”, “*polyphenols*”, “*phytochemical composition*”, “*extraction*”, “*biological activity*”, “*safety*”, “*toxicity*”, “*processing*”, and “*food applications*”. These terms were combined using Boolean operators, according to the search functions of each database. Searches were performed using the Topic field in Web of Science and the corresponding title, abstract, and keyword fields in Scopus. Reference lists of relevant primary studies and review articles were also examined manually to identify additional publications not retrieved through the electronic searches. Particular emphasis was placed on primary studies reporting the extraction, isolation, identification, quantification, biological evaluation, safety, processing, or food utilization of *H. citrina*.

### 2.3. Study Selection

Publications were considered eligible when *H. citrina* Baroni was the experimental plant material or when the study provided directly relevant information on its phytochemical composition, extraction, biological activity, safety, authentication, nutritional properties, processing, or food applications. Studies reporting the extraction, isolation, structural identification, or quantification of flavonoids, phenolic acids, and related polyphenolic constituents were prioritized for the principal phytochemical synthesis. Studies investigating biological or pharmacological activities were also included, with their experimental models and evidence levels considered during interpretation. Relevant research addressing nutrition, processing, toxicity, species authentication, and traditional or food applications was included when it directly supported the objectives of the review.

Studies exclusively investigating other *Hemerocallis* species, including *H. fulva*, *H. flava*, and *H. disticha*, were not treated as direct evidence for *H. citrina*. Such publications were considered only when they provided relevant comparative, taxonomic, methodological, or contextual information, and their findings were clearly distinguished from species-specific evidence. Duplicate records were removed before screening. Publications lacking sufficient information to establish the plant species or experimental material, studies outside the predefined scope, conference abstracts without adequate experimental information, editorials, commentaries, and publications lacking substantive evidence were excluded from the principal synthesis. Review articles were retained primarily for background information and reference tracing, whereas primary research articles were preferentially used to support specific phytochemical, analytical, biological, safety, and food-related claims.

### 2.4. Literature Screening and Data Extraction

The identified records were screened through duplicate removal, title and abstract assessment, and full-text evaluation of potentially relevant publications. Eligibility was assessed with particular attention to botanical identity, plant part, research objective, and relevance to the predefined scope. For each eligible publication, information on publication year, plant species, plant part, cultivar or geographical origin when available, extraction conditions, fractionation and purification procedures, analytical and identification techniques, reported compounds and compound classes, identification level, quantitative information, biological activities and experimental models, processing conditions, safety or toxicity findings, authentication approaches, and food or traditional applications was extracted and organized for narrative synthesis. Particular attention was given to distinguishing evidence obtained directly from *H. citrina* from information derived from other *Hemerocallis* species. When studies investigated multiple species or plant parts, only information specifically attributable to *H. citrina* was incorporated into the corresponding synthesis.

### 2.5. Classification of Phytochemical Identification

To ensure consistency in evaluating phytochemical evidence, reported compounds were classified according to the level of structural confirmation provided by the original studies. Compounds were considered structurally identified when individual constituents had been isolated and their structures were supported by appropriate spectroscopic and/or spectrometric evidence. Compounds identified by chromatographic and mass-spectrometric approaches without isolation were considered tentatively identified when the available evidence was insufficient for definitive structural confirmation. Compounds reported only as components of metabolomic, chromatographic, or aggregate phytochemical profiles were treated as profiling-level evidence rather than as individually characterized constituents. Where available, authentic standards, chromatographic retention behavior, UV–Visible characteristics, MS/MS fragmentation, high-resolution mass spectrometry, NMR spectroscopy, or combinations of these approaches were considered when evaluating identification confidence. Accordingly, detection of a mass-spectral feature alone was not considered equivalent to definitive structural identification. This classification was applied consistently throughout the phytochemical synthesis to distinguish isolated compounds, tentatively identified constituents, and broader profiling results.

### 2.6. Evidence Synthesis and Scope Control

The extracted information was synthesized according to the major objectives of the review, including botanical and nutritional characteristics, flavonoid and phenolic composition, extraction and analytical characterization, biological activities, safety and toxicity, species authentication, processing, and food applications. The phytochemical synthesis prioritized findings directly reported from *H. citrina*, particularly flower buds and flowers. Aggregate metabolomic findings were distinguished from individually characterized compounds, while genomic and biosynthetic information was treated as supporting evidence rather than direct confirmation of individual phytochemicals. Biological activities were interpreted according to experimental model and evidence level, with in vitro, cellular, animal, and human findings distinguished where available.

Other chemical classes, including polysaccharides, anthraquinones, essential oils, proteins, and genomic features, were included only when they provided relevant supporting information for understanding the chemistry, processing, biological activity, safety, authentication, or food utilization of *H. citrina*. Evidence from other *Hemerocallis* species was not used to substantiate species-specific claims unless explicitly identified as comparative or contextual information. The literature search, screening, and evidence synthesis were finalized in September 2026, resulting in 98 references included in the final review.

## 3. Botanical Characteristics, Natural Distribution, and Cultivation

**Figure 2:**
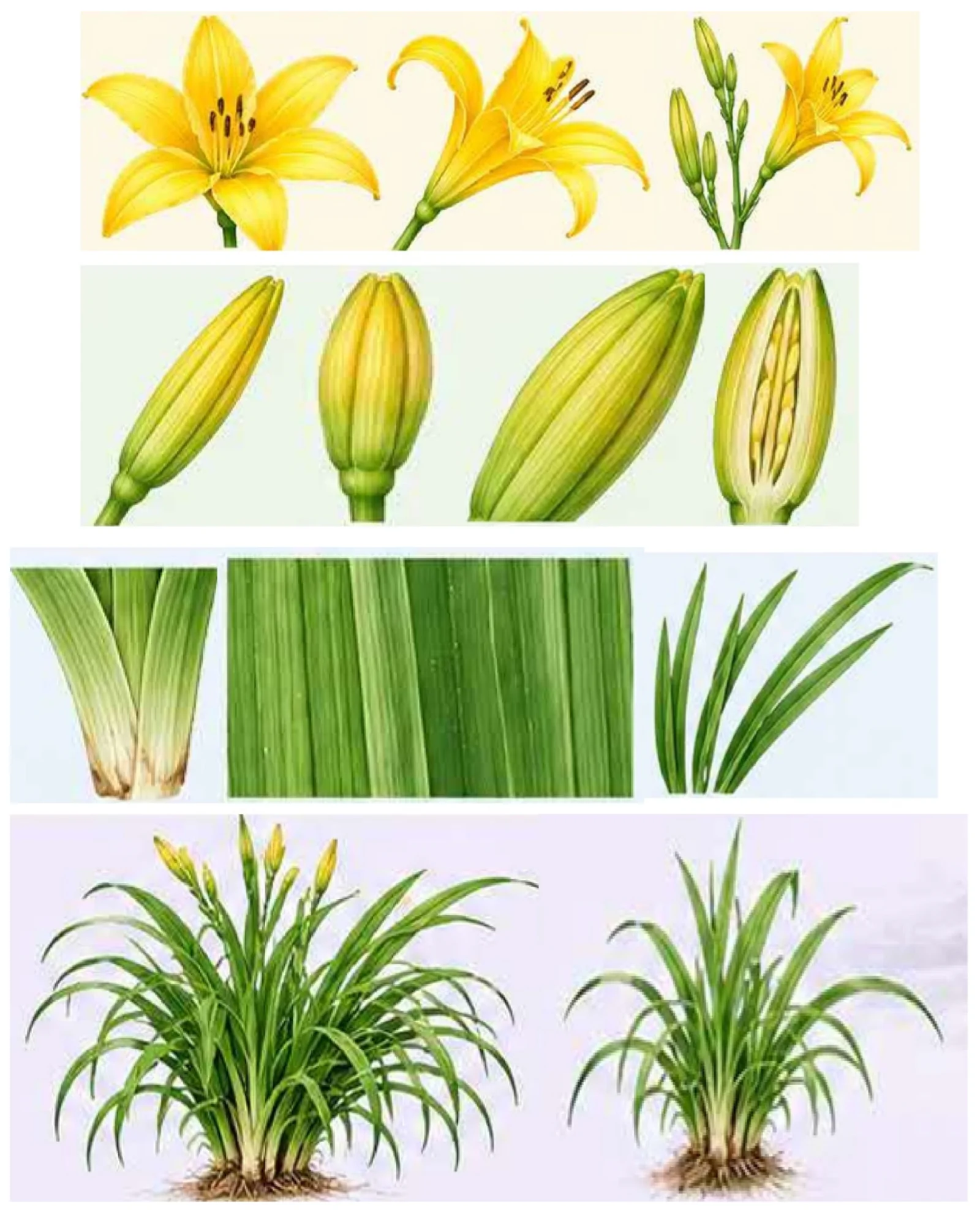
Representative morphological features of *Hemerocallis citrina* Baroni (daylily).

*Hemerocallis citrina* Baroni (*H. citrina*), commonly known as daylily, is a perennial herbaceous plant of the genus *Hemerocallis* (Figure 2). It resembles a lily in appearance but is taxonomically distinct from true lilies. The plant generally reaches 0.3–1.0 m in height and bears relatively short flowering stems with light-yellow flowers. The fruit is a blunt, triangular-oval capsule containing black seeds, and flowering and fruiting extend from May to September. The species prefers light and moisture, tolerates drought well, and generally occurs at elevations below 2000 m [14,15].

### 3.1. Natural Distribution and Geographical Occurrence

*H. citrina* has a long history of use in East Asia as both a food and a medicinal plant. Bi et al. reported China, Japan, and Western Europe as areas of origin and primary distribution [14,15], whereas Jia et al. described the species as occurring wild and cultivated in Asia, including China, Japan, and Korea [12]. Its cultivation in China has been estimated to date back about 2500 years [12]. Natural occurrence should therefore be distinguished from present-day agricultural cultivation, because long-term use as a food and medicinal plant has led to the maintenance of cultivated germplasm alongside naturally occurring populations. Population-level genomic data add evidence on the diversity of *H. citrina* in China. Jia et al. analyzed the plastomes of 65 samples collected from 11 provinces, comprising cultivars and field collections, and found substantial genetic diversity and differentiation among lineages [12]. However, the genetic clusters generally did not correspond to geographic origin. The main exception was one cluster composed mostly of samples from Qidong, Hunan [12].

### 3.2. Current Cultivation and Major Production Regions

*H. citrina* is economically important mainly because its flowers and flower buds are widely used as food and are commonly processed into dried products [6,15]. Different plant parts have also been used for other purposes. Bi et al. reported that roots are used for brewing, leaves for papermaking and weaving, dried flower stems for fuel, and straw as animal feed [15]. Contemporary cultivation is concentrated in China, where the species is grown extensively for its edible flower buds. Jia et al. sampled materials from major production regions in China, including Hunan, Gansu, Shaanxi, Sichuan, Jiangsu, and Shanxi, and also referred to Chongqing and Zhejiang as major production areas. Their sampling localities included Datong (Shanxi), Dali (Shaanxi), Qingyang (Gansu), and Qidong (Hunan) [12]. They also reported considerable genetic diversity among these materials, which underlines the importance of regional germplasm resources for conservation, breeding, and utilization [12].

The composition of harvested material can also vary with germplasm [12] and developmental stage. Mu et al. showed that the nutritional and bioactive profiles of *H. citrina* flowers changed across the initial, full-bloom, and final flowering stages, indicating that harvest timing influences the characteristics of the material [11]. Post-harvest processing is another source of variation. Ding et al. investigated effective diffusivities and energy consumption during microwave drying of daylily, which shows the importance of drying conditions for dried products [6]. Germplasm, developmental stage, harvest conditions, and processing should therefore be considered when comparing results from different studies.

### 3.3. Traditional Food and Medicinal Utilization

The flowers and flower buds are the principal edible parts of *H. citrina* and have long been consumed as a vegetable. The plant also has a long history of traditional medicinal use. Previous studies have summarized folk-medicine applications related to swelling, pain, bleeding, blood pressure, and abscesses, and have noted its traditional sedative use [14,15]. A systematic review by Matraszek-Gawron et al. compiled literature data on the antidepressant activity of *Hemerocallis* species, including *H. citrina* and *H. fulva* [14]. Because the present review focuses on flavonoids and phenolic/polyphenolic compounds, these uses are treated as supporting context. Drying is important for *H. citrina* because dried products are widely used and can be stored stably. Microwave-drying studies have examined moisture transfer, effective diffusivity, and energy consumption in daylily [6]. These processing factors matter when interpreting phytochemical studies, because extraction and analytical results may differ between fresh, dried, and otherwise processed materials.

### 3.4. Botanical Identification and Species Authentication

A practical challenge in using *H. citrina* is its similarity to *Hemerocallis fulva* (L.) L. Bi et al. described the two as difficult to distinguish, noted that the main botanical difference lies in the surface and color of the fruit, and reported that poisoning from ingestion is common [15]. Species identification is therefore important for safe use. Chemical and molecular approaches can complement botanical identification. Another study established an HPLC-Q-TOF-MS strategy for the systematic identification of metabolites in *H. citrina*, which provides chemical information that may support species characterization [7]. Jia et al. showed that plastome variation can resolve maternal lineages and provide markers for germplasm identification [12]. Bi et al. proposed building an identification system based on polysaccharide characteristics [15].

In summary, the wide cultivation, diverse germplasm, and long history of food and medicinal use make *H. citrina* an important edible and medicinal plant. Differences in germplasm, developmental stage, processing, and species identity should be considered when interpreting the phytochemical and biological findings discussed in the following sections.

## 4. Nutritional Composition and Dietary Value

Fresh *Hemerocallis citrina* Baroni flowers have been reported to contain substantial amounts of macronutrients and micronutrients. According to the nutritional data summarized by Bi et al. [15], the edible portion of fresh *H. citrina* flowers contains 19.4 g protein per 100 g, together with 34.9 g carbohydrate, 1.4 g fat, and 4.0 g ash. The same source reports that the protein content of *H. citrina* flowers is approximately 21.6-fold that of tomato and 14.9-fold that of cabbage, based on the corresponding comparative nutritional data reported in that source [15]. These ratios are therefore presented here as source-reported comparisons rather than as independent measurements from the present review.

The reported nutritional profile also includes carotene, thiamin, riboflavin, niacin, vitamin C, and several essential minerals, including calcium, phosphorus, potassium, magnesium, iron, zinc, selenium, copper, and manganese [15]. The complete nutritional composition is presented in Table 1. Because nutritional composition can vary with cultivar, developmental stage, growing conditions, processing, and the compositional basis used for comparison, the values in Table 1 should be interpreted specifically as reported values per 100 g edible portion of fresh *H. citrina* flowers, unless otherwise indicated by the cited source [15].

## 5. Phytochemical Composition of *Hemerocallis citrina* Baroni: Flavonoids and Phenolic Compounds

### 5.1. Flavonoids and Phenolic Compounds: Brief Background

Flavonoids are a major class of polyphenolic compounds characterized by a C6-C3-C6 carbon framework consisting of two aromatic rings connected through a three-carbon heterocyclic C-ring [16]. Differences in hydroxylation, oxidation, and substitution patterns give rise to substantial structural diversity, and flavonoids are commonly classified into major subclasses including flavones, flavonols, flavanols, flavanones, isoflavones, and anthocyanidins [17]. In plants, flavonoids participate in pigmentation, protection against environmental stresses, plant–microbe interactions, and antioxidant defense, while their structural characteristics can influence their physicochemical and biological properties [18,19,20,21,22,23].

Phenolic acids represent another important group of plant phenolics and are broadly categorized into hydroxybenzoic acid and hydroxycinnamic acid derivatives according to their carbon skeletons. They may occur as free compounds or in conjugated forms such as esters, amides, and glycosides, with these structural forms influencing their stability, solubility, and biological properties [19,24,25]. Phenolic acids and flavonoids are therefore important constituents of plant-derived foods and medicinal plants and have received considerable attention because of their reported antioxidant and other biological activities [19,21,22,24,25].

In *Hemerocallis citrina* Baroni, these phenolic constituents have attracted increasing attention because phytochemical investigations have revealed considerable structural diversity, particularly in the flowers and flower buds. Reported constituents include flavonoid glycosides, phenolic acids, hydroxycinnamate derivatives, lignans, and other phenolic metabolites. The following subsection therefore focuses specifically on the compounds that have been isolated, identified, or tentatively detected in *H. citrina*, with particular emphasis on flower buds and flowers.

### 5.2. Flavonoids and Phenolic Compounds Identified in Hemerocallis citrina Baroni

Several recent phytochemical investigations have substantially expanded the known chemical diversity of *Hemerocallis citrina* Baroni. Ma et al. conducted a detailed phytochemical investigation of the flower buds of *H. citrina*, resulting in the isolation of 21 secondary metabolites, with the isolated constituents predominantly represented by polyphenolic and flavonoid-related compounds. Among these isolates, three previously undescribed compounds, namely hemeroquinone A (**1**), hemeroquinone B (**2**), and hemerophenan A (**3**), were reported for the first time from the genus *Hemerocallis*. In addition to these new compounds, several previously known metabolites, including 2,4-dihydroxy-6-(4-hydroxyphenethyl)-methyl benzoate (**4**), 3-methyl-2,4-dihydroxy-6-(4-hydroxyphenethyl)-methyl benzoate (**5**), (+)-pinoresinol (**6**), (+)-epipinoresinol (**7**), pinoresinol-4-*O*-β-D-glucoside (**8**), and cinnamic acid (**9**), were also obtained from the flower buds. Collectively, these isolates represent a structurally diverse group of phenolic and related polyphenolic constituents, further demonstrating the rich secondary-metabolite profile of *H. citrina* flower buds. The biological activities of the isolated compounds were also investigated using LPS-stimulated BV2 microglial cells. Compounds **1–7** exhibited inhibitory effects on nitric oxide (NO) production, suggesting potential anti-neuroinflammatory activity. Subsequent mechanistic investigations demonstrated that compound **1** attenuated neuroinflammatory responses through negative regulation of the MAPK and NF-κB signaling pathways [26]. These findings not only expanded the known chemical diversity of *H. citrina* but also provided evidence linking several flower-bud constituents with anti-neuroinflammatory activity.

The chemical diversity of *H. citrina* was further extended by investigation of its stems and leaves. Li et al. conducted a comprehensive phytochemical analysis of the stems and leaves of *H. citrina* Baroni and isolated four previously undescribed phenolic compounds, designated hemerodiphenylethanes A–D (**10–13**). Their structures were elucidated using high-resolution electrospray ionization mass spectrometry (HR-ESI-MS), one-dimensional and two-dimensional nuclear magnetic resonance (1D/2D-NMR) spectroscopy, and electronic circular dichroism (ECD) computational analysis [27]. This integrated spectroscopic and computational approach enabled structural and stereochemical characterization of the newly identified metabolites. Notably, compounds **10** and **12** were reported from the *Hemerocallis* genus for the first time, whereas the remaining compounds represented their first isolation from *H. citrina*. Beyond their structural novelty, these phenolic constituents exhibited free-radical-scavenging activities in DPPH and ABTS^+ assays, with their antioxidant potential further evaluated through determination of IC_50_ values [27]. These findings indicate that, in addition to flower buds, the stems and leaves of *H. citrina* also contain structurally diverse phenolic metabolites with measurable antioxidant activity.

Complementary to isolation-based investigations, Wang et al. employed ultra-performance liquid chromatography coupled with photodiode array and mass spectrometric detection (UPLC-PDA/MS) to characterize the phenolic and flavonoid composition of an ethyl acetate fraction of dried *H. citrina* flowers [28]. Comparison with authentic reference standards enabled the unambiguous confirmation of several flavonoids, including hyperoside, isoquercitrin, astragalin, and rutin. Additional constituents were identified or tentatively assigned based on chromatographic behavior, UV absorption characteristics, mass-fragmentation patterns, and comparison with previously reported data [28]. These compounds included 5-*O*-caffeoylquinic acid (chlorogenic acid) (**14**), gallic acid (**15**), 4-*O*-*p*-coumaroylquinic acid (**16**), 1-cyclohexene-1-carboxylic acid (**17**), quercetin 3,7-di-*O*-β-D-glucopyranoside (**18**), phloretin 2′-*O*-β-D-xylopyranosyl-(1→6)-β-D-glucopyranoside (**19**), kaempferol-3-*O*-galactoside (**20**), kaempferol 3-*O*-rutinoside (**21**), astragalin (**22**), and isorhamnetin 3-*O*-glucoside (**23**). These findings demonstrate the chemical complexity of *H. citrina* flowers and highlight the coexistence of phenolic acids, flavonol glycosides, caffeoylquinic acid derivatives, and other polyphenolic constituents [28].

Beyond conventional isolation procedures, targeted and untargeted chromatographic–mass-spectrometric approaches have further expanded the known flavonoid and phenolic-acid profiles of *H. citrina*. Ma et al. developed an ultra-high-performance liquid chromatography coupled with triple-quadrupole mass spectrometry (UHPLC-QQQ-MS/MS) approach for the comprehensive characterization of flavonoid and phenolic constituents in *H. citrina* flowers. Diagnostic ions corresponding to different flavonoid aglycones, including quercetin, kaempferol, isorhamnetin, myricetin, syringetin, laricitrin, galangin, and naringenin, were used to facilitate the characterization of their derivatives [29]. The study detected a total of 58 flavonoid compounds, with kaempferol-, quercetin-, and isorhamnetin-derived compounds representing major groups. Ten flavonoids were accurately identified through comparison with authentic reference standards. These included rutin, quercetin-3-*O*-α-L-arabinopyranoside (**24**), kaempferol-3-*O*-β-D-galactopyranoside (**25**), kaempferol-3-*O*-β-D-glucopyranoside (**26**), isorhamnetin-3-*O*-β-D-galactopyranoside (**27**), isorhamnetin-3-*O*-β-D-glucopyranoside (**28**), kaempferol-3-*O*-[α-L-rhamnopyranosyl-(1→6)]-[α-L-rhamnopyranosyl-(1→2)]-β-D-glucopyranoside (**29**), isorhamnetin-3-*O*-[α-L-rhamnopyranosyl-(1→6)]-β-D-glucopyranoside (**30**), quercetin-3-*O*-β-D-galactopyranoside (hyperoside) (**31**), and quercetin-3-*O*-β-D-glucopyranoside (isoquercitrin) (**32**) [29]. The structural diversity of these compounds illustrates the extensive glycosylation patterns associated with the major flavonoid aglycones in *H. citrina* flowers.

In addition to flavonoids, several phenolic-acid derivatives were identified in the same investigation, including crypto-chlorogenic acid (4-*O*-caffeoylquinic acid) (**33**), 3-*O*-(Z)-*p*-coumaroylquinic acid (**34**), 3-*O*-(E)-*p*-coumaroylquinic acid (**35**), 4-*O*-(E)-*p*-coumaroylquinic acid (**36**), 4-*O*-(Z)-*p*-coumaroylquinic acid (**37**), and 5-*O*-(E)-*p*-coumaroylquinic acid (**38**) [29]. These findings demonstrate that the phenolic profile of *H. citrina* flowers extends beyond simple flavonoid glycosides to include structurally diverse hydroxycinnamate and quinic-acid derivatives.

Further analytical evidence for individual phenolic constituents was provided by the detection of 4-hydroxycinnamic acid (**39**) in *H. citrina* flowers using UPLC-Q-TOF-MS/MS [29,30,31,32,33]. The compound was verified through comparison of its accurate mass-to-charge ratio (*m*/*z*) with that of a reference standard [29,30,31,32,33]. Its inclusion as compound **39** in Table 2 reflects its status as an individual phenolic constituent rather than an aggregate profiling result. Together with the caffeoylquinic acid and *p*-coumaroylquinic acid derivatives described above, the detection of 4-hydroxycinnamic acid further supports the occurrence of diverse hydroxycinnamate-related metabolites in *H. citrina* flowers.

**Figure 3:**
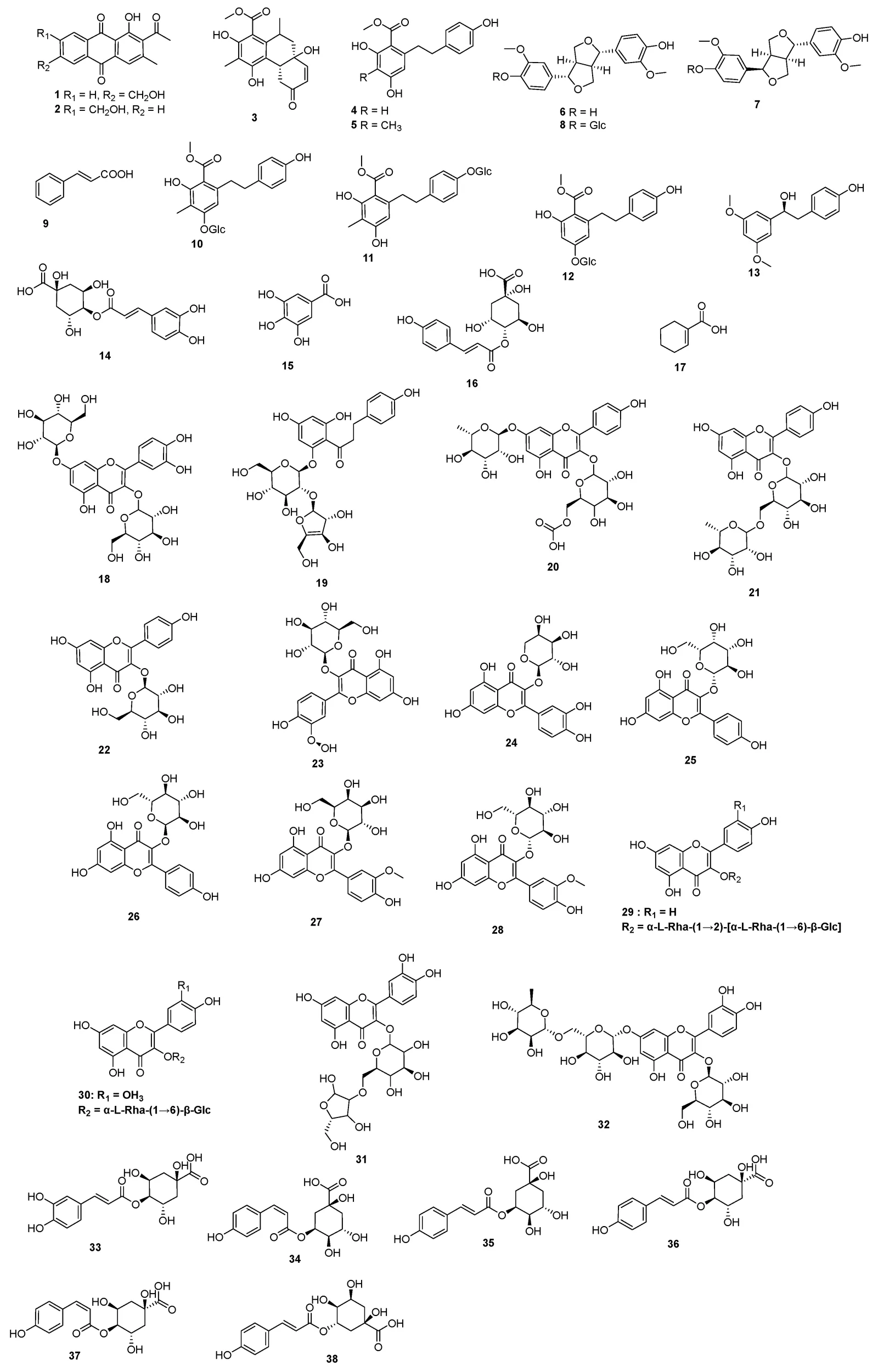
Chemical structures of phenolic and flavonoid compounds **1–38** from *Hemerocallis citrina* Baroni.

Beyond these individually reported compounds, broader metabolomic investigations have revealed an even greater diversity of phenolic constituents in *H. citrina*. The application of product ion filtration (PIF), neutral loss filtration (NLF), and the Global Natural Products Social Molecular Networking (GNPS) database enabled the preliminary annotation of an additional 35 potential flavonoid compounds in *H. citrina* flowers [29]. These putatively annotated metabolites were primarily derivatives containing kaempferol, quercetin, isorhamnetin, myricetin, syringetin, laricitrin, galangin, and naringenin as their respective aglycones. Rutin was reported as the most abundant flavonoid among the detected constituents, highlighting its potential importance within the phenolic profile of *H. citrina* flowers [29]. Because these compounds were reported as putative annotations rather than individually confirmed structures, they are not assigned separate compound numbers in Table 2, Figure 3.

Further evidence for the richness of the phenolic composition of *H. citrina* was provided by Mu et al., who identified 6 phenolic acids and 71 flavonoids in flower material [30]. Their study also investigated the behavior and functionality of these polyphenols in a soybean-protein-isolate emulsion system. Encapsulation within the emulsion matrix enhanced the release and stability of the polyphenolic constituents and improved their antioxidant efficacy during simulated intestinal digestion [30]. Flavonoids, in particular, exhibited enhanced release and antioxidant activity, suggesting that suitable delivery or food-matrix systems may improve the functional availability of *H. citrina* polyphenols. Molecular docking analysis further indicated favorable interactions for several constituents, with quercetin showing the most favorable binding affinity, followed by naringenin, isorhamnetin, 4-hydroxybenzoic acid, isoquercitrin, and rutin [30]. These findings provide useful evidence linking the chemical composition of *H. citrina* with potential functional applications of its polyphenolic constituents, particularly in antioxidant-rich foods and nutraceutical systems. Because the reported numbers represent aggregate phytochemical profiling rather than individually characterized compounds, these findings are summarized separately in Table 3.

In addition to phytochemical investigations, genomic studies have provided valuable insights into the biosynthetic potential underlying flavonoid accumulation in *H. citrina*. Qing et al. generated a high-quality chromosome-level reference genome of *H. citrina* Baroni, providing an important genomic resource for investigating the evolutionary history and specialized metabolism of this species. The assembled genome comprised 3.77 Gb and contained 54,295 protein-coding genes. Comparative genome-evolution analyses indicated a recent whole-genome duplication event approximately 15.73 million years ago [31]. Using this reference genome, the authors identified 20 genes potentially involved in rutin biosynthesis, providing a molecular framework for understanding the formation and accumulation of this important flavonoid glycoside [31,32]. Integrated metabolomic and genomic analyses also found no evidence for colchicine or its associated biosynthetic pathway in *H. citrina* [31,32]. These genomic and biosynthetic findings are summarized separately in Table 3 because they provide contextual molecular evidence rather than individual compound-level phytochemical identifications.

Similarly, Liu et al. employed HPLC-Q-TOF-MS together with a multi-strategy screening workflow incorporating non-targeted, accurate-targeted, and extensive-targeted approaches to comprehensively profile metabolites in commercially available *H. citrina* flower buds. Using this integrated analytical strategy, 44 flavonoid compounds were detected. Among these, rutin, kaempferol, and quercetin were unambiguously identified through direct comparison with authentic reference standards based on accurate mass measurements and characteristic fragmentation patterns. The remaining compounds were tentatively assigned primarily as derivatives of rutin, kaempferol, and quercetin through interpretation of their MS/MS spectra and diagnostic fragment ions [34]. These findings provide additional evidence for the predominance of flavonol-type metabolites in *H. citrina* flower buds and demonstrate that advanced mass-spectrometric profiling can reveal substantially broader flavonoid diversity than that accessible through conventional compound-isolation approaches alone. Because the reported 44 flavonoids represent an aggregate profiling result, rather than 44 individually confirmed compounds presented in the current review, this finding is summarized separately in Table 3.

Taken together, the findings from isolation studies, targeted quantitative analyses, high-resolution mass spectrometry, metabolomic investigations, and genomic studies reveal a diverse polyphenolic profile in *H. citrina*. The individually reported constituents encompass structurally diverse flavonoids, flavonoid glycosides, phenolic acids, hydroxycinnamate derivatives, lignans, and other phenolic or related polyphenolic metabolites. Particularly noteworthy is the repeated identification of quercetin, kaempferol and isorhamnetin-derived compounds, which appear to constitute important components of the flavonoid profile of the flowers and flower buds. At the same time, the discovery of newly isolated compounds and the increasing number of tentatively annotated metabolites indicate that the currently characterized chemical diversity of *H. citrina* may represent only part of its total specialized-metabolite repertoire.

Importantly, compound-level identification should be distinguished from aggregate profiling and tentative annotation. Compounds presented in Table 2 represent individual constituents that have been isolated or analytically identified, whereas compounds reported only as putative annotations or as part of aggregate metabolomic counts are not assigned individual numbers. Such putative assignments may require further confirmation using authentic standards, NMR spectroscopy, or other orthogonal structural techniques. Table 3 therefore complements Table 2 by summarizing aggregate phytochemical profiling and genomic/biosynthetic evidence rather than individual compounds. This distinction provides a clearer representation of the current level of phytochemical evidence available for *H. citrina* and avoids treating tentative annotations, aggregate metabolite counts, and genomic findings as equivalent to definitive compound-level identification.

## 6. Extraction Procedures for Flavonoids and Polyphenols from *Hemerocallis citrina* Baroni

Various extraction strategies have been developed to recover flavonoids, phenolic acids, phenylpropanoids, and other polyphenolic constituents from *Hemerocallis citrina* flower buds and flowers. The reported approaches include conventional solvent extraction, aqueous-alcoholic extraction, ultrasound-assisted extraction, and subsequent solvent partitioning or chromatographic fractionation [26,27,28,29,30,31,32,33,34,35,36,37,38,39]. The selection of solvent composition, extraction time, temperature, and downstream fractionation can substantially influence the recovery and composition of the resulting phenolic-rich fractions. The principal extraction and analytical approaches reported for flavonoids and phenolic compounds from *H. citrina* are summarized in Table 4.

For the isolation of individual phenolic constituents, conventional solvent extraction followed by solvent partitioning and chromatographic purification has been employed. In one representative study, *H. citrina* flower buds were extracted repeatedly with 80% ethanol by cold-dipping, followed by concentration and fractionation using AB-8 macroporous resin chromatography. Phenylpropanoid- and flavonoid-enriched fractions were subsequently obtained and characterized by UHPLC–Q-TOF-MS, with 13 phenylpropanoids and 21 flavonoids reported from the corresponding fractions [8]. For structurally characterized compounds, chromatographic purification combined with HR-ESI-MS, one- and two-dimensional NMR spectroscopy, and electronic circular dichroism (ECD) analysis was used to establish the structures of isolated constituents [26,27].

Ultrasound-assisted extraction has also been widely applied to improve the recovery of phenolic constituents from *H. citrina*. Wang et al. extracted powdered dried flowers three times with 70% ethanol under ultrasonic conditions (40 min, 55 °C), followed by concentration and sequential partitioning with *n*-hexane, ethyl acetate, and *n*-butanol. The ethyl acetate fraction was subsequently analyzed by UPLC-PDA/MS and contained flavonoids and phenolic acids, including hyperoside, isoquercitrin, astragalin, rutin, gallic acid, and chlorogenic acid [28]. These results demonstrate the utility of aqueous ethanol combined with ultrasound-assisted extraction and solvent partitioning for obtaining phenolic-rich fractions.

More recently, simplified ultrasound-assisted extraction using 50% methanol for 30 min at room temperature has been employed for comprehensive profiling of *H. citrina* flowers. UHPLC–Q-TOF-MS/MS and UHPLC–QQQ-MS/MS enabled the characterization and quantitative determination of numerous flavonoids and phenylpropanoid constituents, including caffeoyl- and *p*-coumaroylquinic acid derivatives [29,30,31,32,33]. Similarly, methanolic extraction assisted by ultrasonication (40 kHz, 30 min) was applied to dried flower buds from Sichuan, China, followed by UPLC–Q-TOF-MS/MS analysis and non-target, accurate-target, and extensive-target screening [33]. These studies illustrate the increasing integration of ultrasound-assisted extraction with high-resolution mass spectrometry for broad phytochemical characterization [34,35,36].

Comparative extraction approaches have further demonstrated the influence of solvent composition and extraction technology on phenolic recovery. Ultrasound-assisted water and ethanol extraction and enzyme-assisted extraction have been investigated for *H. citrina* flowers, with 50% ethanol-assisted ultrasonication providing favorable recovery of total phenolic compounds and antioxidant-associated constituents [37]. In another study, hydroalcoholic extracts prepared using different ethanol concentrations were compared, and the 75% ethanol extract was selected for subsequent chemical and biological investigations [36]. For phenylpropanoid-rich fractions, water extraction followed by ethanol-based fractionation has also been employed. A 35% ethanol-eluted fraction obtained from a water extract was used for subsequent characterization of major phenylpropanoid constituents, including 3-O-*p*-coumaroylquinic acid and 4-O-*p*-coumaroylquinic acid [35].

Solvent partitioning and chromatographic fractionation can provide additional enrichment of target phenolic constituents following the initial extraction step. Sequential partitioning with solvents of different polarity, particularly ethyl acetate and *n*-butanol, has been used to separate phenolic-rich fractions from crude aqueous-alcoholic extracts [28]. Macroporous resin chromatography has likewise been used to enrich phenylpropanoid- and flavonoid-containing fractions before subsequent chromatographic and mass-spectrometric characterization [8]. For individual compound isolation, further column-chromatographic purification and spectroscopic characterization are required to distinguish isolated compounds from compounds detected only at the profiling level [26].

The analytical approaches used alongside extraction have progressively shifted from conventional chromatographic analysis toward high-resolution mass spectrometry and multidimensional structural characterization. UHPLC–Q-TOF-MS/MS, UHPLC–QQQ-MS/MS, UPLC-PDA/MS, and related techniques have enabled broad qualitative and quantitative profiling, whereas HR-ESI-MS, NMR, and ECD have provided complementary evidence for the structural characterization of isolated constituents [8,26,28,29,33]. Importantly, analytical detection of a compound should be distinguished from definitive structural characterization or isolation; therefore, the revised Table 3 identifies the analytical approaches used in each study without implying that all detected constituents were individually isolated.

Studies focused primarily on essential-oil hydrodistillation and volatile constituents are not included in this extraction section because essential oils represent a chemically distinct fraction from the flavonoid and phenolic/polyphenolic constituents that are the focus of this review. Accordingly, the essential-oil study [38] has been removed from Table 3 and from the core extraction discussion and is considered separately where relevant to the biological or compositional characteristics of *H. citrina*. Likewise, studies primarily investigating antidepressant, cognitive, or other pharmacological effects [39] are not presented here as extraction studies; their biological findings are discussed in the corresponding sections on biological activities. Where extraction procedures from pharmacological studies are relevant to the preparation of flavonoid- or phenolic-containing extracts, only the pertinent extraction information is retained in Table 4 [36].

Overall, the available evidence indicates that aqueous ethanol and methanol, particularly when combined with ultrasound-assisted extraction, are commonly used for recovering flavonoids and phenolic compounds from *H. citrina*. Solvent polarity, extraction temperature and duration, ultrasound treatment, and subsequent fractionation can influence both extraction efficiency and the composition of the resulting fractions. However, differences in plant material, geographical origin, preprocessing, solvent-to-material ratio, extraction conditions, and analytical platforms make direct comparison of extraction yields across studies difficult. The extraction procedures, plant materials, fractionation strategies, and analytical methods reported for *H. citrina* flavonoids and phenolic/polyphenolic compounds are summarized in Table 4.

## 7. Biological Activities of Polyphenols, Flavonoids, and Related Constituents from *Hemerocallis citrina* Baroni

**Figure 4:**
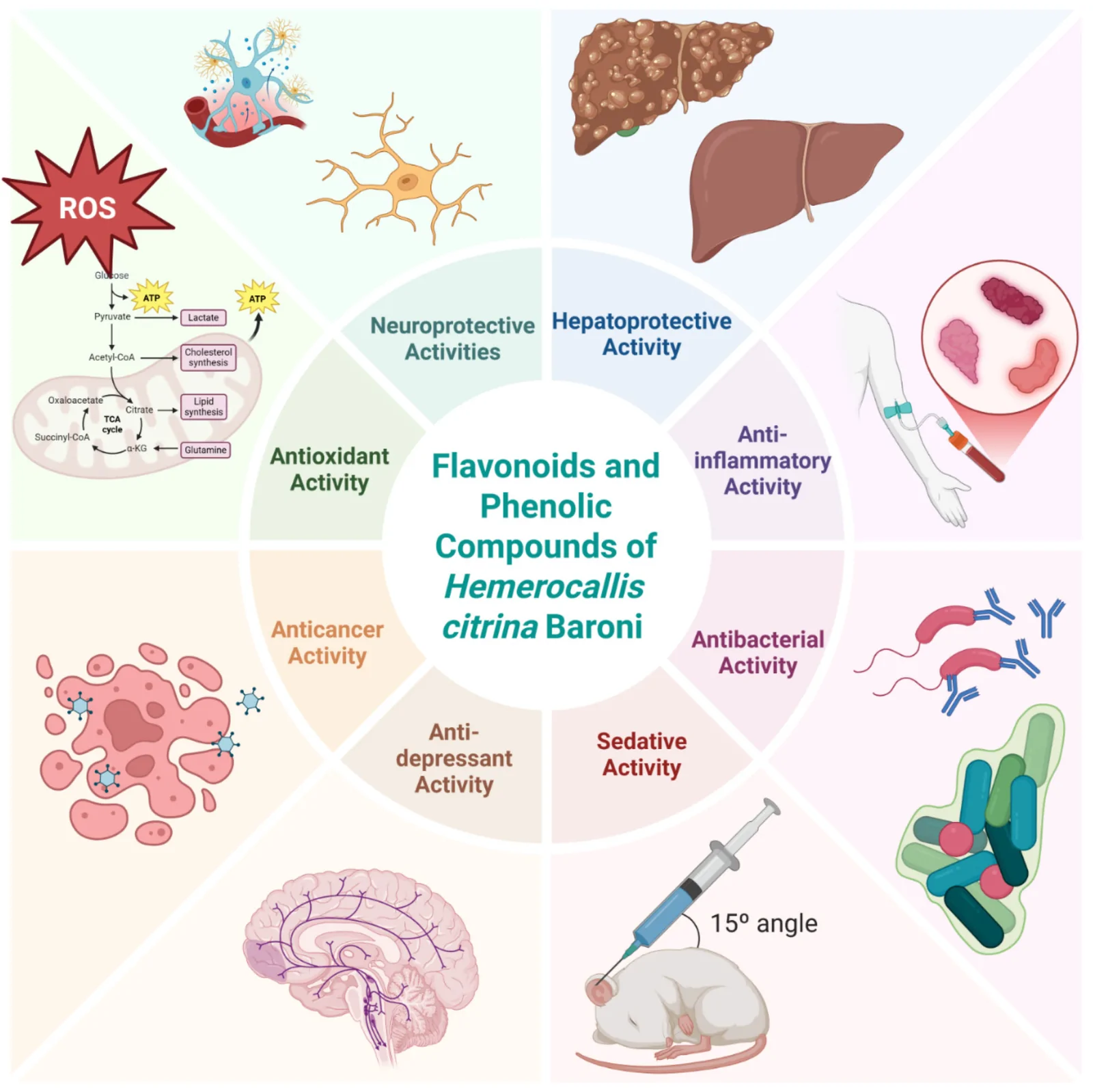
Biological activities of the flavonoids and polyphenols of *Hemerocallis citrina* Baroni.

The edible flower buds of *Hemerocallis citrina* Baroni are rich in polyphenols (especially phenolic acids and flavonoids), polysaccharides, anthraquinones, and other secondary metabolites. These constituents are responsible for a broad spectrum of pharmacological activities that have been extensively investigated in both traditional Chinese medicine and modern pharmacological studies Figure 4. The following sections provide a comprehensive overview of the major biological activities, incorporating mechanistic details, experimental evidence, and supporting references. These biological activities are discussed in the following sections and summarized in Table 5.

### 7.1. Antioxidant Activity

Oxidative stress caused by the excessive accumulation of reactive oxygen species (ROS) and free radicals is a primary driver of cellular damage, accelerated aging, and the pathogenesis of numerous chronic diseases. Extracts of *H. citrina* flower buds exhibit potent antioxidant capacity attributable to their high content of flavonoids, phenolic acids, and polysaccharides. Previous studies have reported that both fresh and dried daylily extracts scavenged DPPH radicals at rates of 17.1 ± 0.6% and 22.5 ± 0.5%, respectively [40,41]. Hao et al. compared four extraction approaches—ultrasonic-assisted water extraction, ultrasonic-assisted 50% ethanol extraction, enzyme-assisted water extraction, and enzyme-assisted ethanol extraction to evaluate the antioxidant potential of *Hemerocallis citrina* extracts [37]. Among these, the ultrasonic-assisted 50% ethanol extract (UE) exhibited the highest total phenolic content (130.05 mg/100 g dry weight) and the strongest antioxidant activity. Consistent with its higher phenolic content, the UE extract showed the greatest DPPH radical-scavenging activity (7.75 mg Trolox/g dry weight), ferric-reducing antioxidant power (FRAP; 14.54 mg Trolox/g dry weight), and ABTS radical-scavenging activity (15.37 mg Trolox/g dry weight), significantly outperforming the other extraction treatments. The authors further observed a positive relationship between phenolic content and DPPH-scavenging capacity, suggesting that the enhanced antioxidant activity was closely associated with the greater recovery of phenolic compounds through ultrasonic-assisted extraction. These findings highlight 50% ethanol-assisted ultrasonication as an effective strategy for obtaining antioxidant-rich *H. citrina* extracts [37]. Modern studies have elucidated molecular mechanisms. The ethyl acetate fraction of dried flowers (HCEA) not only enhanced total antioxidant capacity (T-AOC) and the enzymatic activities of superoxide dismutase (SOD) and catalase (CAT) in BRL-3A cells, but also upregulated key genes of the Nrf2 signaling pathway, including *Ampk*, *Erk*, *P38*, *Nrf2*, *Sod*, *Gclc*, *Gclm*, and *Ho-1*. Encapsulation of daylily polyphenols in soybean-protein-isolate emulsions further improved their stability, intestinal release, and antioxidant efficacy during simulated digestion, with quercetin, naringenin, isorhamnetin, isoquercitrin, and rutin identified as major contributors via molecular docking [30]. Newly isolated phenolic compounds from stems and leaves also exhibited significant DPPH and ABTS^+^ radical-scavenging activity [27]. Collectively, these findings establish that the polyphenolic and polysaccharide constituents of *H. citrina* provide robust protection against oxidative damage through both direct radical scavenging and upregulation of endogenous antioxidant defense systems.

### 7.2. Anti-Tumor Activity

Multiple classes of compounds in *Hemerocallis citrina*, including anthraquinones, colchicine-related alkaloids, polysaccharides, and proteins, have demonstrated anti-tumor potential. Studies reported that aqueous extracts inhibited the proliferation of human dermal fibroblasts in a concentration-dependent manner by interfering with the microtubule protein system, an effect reminiscent of colchicine action [41,42]. Cichewicz et al. isolated novel anthraquinones from root methanol extracts that suppressed the proliferation of breast and lung cancer cells; the cytotoxicity was further enhanced by vitamins C and E, although topoisomerase activity was not inhibited [43]. More recent investigations have provided deeper mechanistic insights. You et al. purified a low-toxicity protein (HcBP) possessing peroxidase activity. HcBP downregulated pyruvate kinase M2 (PKM2) protein and mRNA levels, suppressed aerobic glycolysis-related genes (*PDK*, *LDHA*, *GLUT1*), elevated intracellular ROS, and activated the mitochondrial apoptotic pathway (increased Cytochrome C, BAX, Caspase-9, and Caspase-3; decreased BCL-2), thereby inhibiting proliferation of HepG2 and Bel-7402 hepatocellular carcinoma cells [42]. Flavonoids from *Hemerocallis citrina* have demonstrated anticancer potential by inducing cell-cycle arrest and promoting mitochondrial-mediated apoptosis, while also suppressing the Wnt/β-catenin signaling pathway. These effects are associated with modulation of key regulatory proteins involved in cell-cycle progression and apoptosis, suggesting that the flavonoid constituents may contribute to the antitumor properties of *Hemerocallis citrina* [43].

### 7.3. Anti-Depressant Activity

Depression is a multifactorial neuropsychiatric disorder involving complex and interconnected mechanisms, including disturbances in monoaminergic neurotransmission, hyperactivity of the hypothalamic–pituitary–adrenal (HPA) axis, neuroinflammation, and alterations in the gut–brain axis and intestinal microbiota. *Hemerocallis citrina* Baroni has a long history of traditional use for alleviating depressive symptoms, and accumulating pharmacological evidence supports its antidepressant potential. In particular, flavonoids and phenylpropanoid/phenolic constituents have emerged as important contributors to the antidepressant effects of *H. citrina*, with compounds such as rutin and hesperidin demonstrating significant activity in experimental models. The antidepressant-like effects of hydroalcoholic extracts of Hemerocallis citrina prepared with different ethanol concentrations (0–100%) have also been investigated [36,44]. Among them, the 75% ethanol extract (HCE75) exhibited the highest flavonoid content and the strongest antidepressant-like activity in the tail suspension test, without producing nonspecific locomotor stimulation in the open-field test. HPLC analysis identified rutin and hesperidin as the predominant flavonoids in HCE75, accounting for approximately 75% and 21.5% of its flavonoid composition, respectively. Oral administration of HCE75 at 400 mg/kg significantly increased brain serotonin (5-HT) and dopamine (DA) levels, supporting the involvement of serotonergic and dopaminergic neurotransmission in its antidepressant-like effects. [36,44]. Urinary metabolomics revealed that the extracts modulated phenylalanine, glutamate, and energy metabolism pathways [39].

Subsequent studies have confirmed and expanded these findings. Yi et al. reported that ethanol extracts reversed CORT-induced depression-like behaviors and upregulated BDNF and TrkB expression in the prefrontal cortex and hippocampus via the BDNF-TrkB pathway [45]. Xu et al. further showed cognitive improvement alongside antidepressant effects, linked to reduced oxidative stress and elevated monoamines and BDNF [39]. Liu et al. observed downregulation of IL-1β, IL-6, and TNF-α and reduced indoleamine 2,3-dioxygenase (IDO) activity in CUMS rats [46]. Ma et al. demonstrated that enriched phenylpropanoid and flavonoid fractions lowered corticosterone and pro-inflammatory cytokines [8]. Liu et al. additionally showed that water extract plus rutin modulated the gut microbiota (*Bacteroides* increased, *Desulfovibrio* decreased), thereby influencing the microbiota–gut–brain axis [23]. These multi-target actions, monoamine elevation, neurotrophic support, anti-inflammation, HPA-axis regulation, and microbiota modulation, collectively underlie the robust antidepressant efficacy of daylily polyphenols.

### 7.4. Sleep-Improving and Sedative Activity

Adequate sleep is essential for physiological recovery and emotional well-being. Early studies reported sleep-promoting effects of hydroalcoholic and ethanol extracts of *Hemerocallis citrina* in animal models [47,48]. Gu et al. further demonstrated that an ethanol extract of *H. citrina* produced antidepressant-like effects without altering spontaneous locomotor activity, while enhancing 5-HT, norepinephrine, and dopamine levels in specific brain regions [49]. More recent studies using *Drosophila melanogaster* provided direct evidence for sleep-promoting activity: *H. citrina* extracts reduced nighttime locomotor activity and significantly prolonged total sleep time in a caffeine-induced insomnia model [50]. Targeted screening implicated compounds including quercetin, luteolin, kaempferol, caffeic acid, and nicotinic acid in the regulation of sleep-related pathways [50]. Integrated activity monitoring, metabolomics, targeted screening, and transcriptomic analyses further confirmed that *H. citrina* extracts enhanced sleep and suggested a multi-component, multi-target mechanism underlying these effects [51].

### 7.5. Antimicrobial Activity

Anthraquinones, flavonoids, and essential-oil components contribute to antimicrobial effects [52]. Vorobets et al. reported that water-ethanolic extracts of *Hemerocallis fulva* flowers and leaves, rich in polyphenols and flavonoids, inhibited all tested bacteria (*Bacillus subtilis*, *Staphylococcus albus*, *Escherichia coli*, and *Micrococcus luteus*) as well as the yeasts *Candida pseudotropicalis* and *Candida parapsilosis* in agar diffusion assays [53]. Szewczyk et al. found that steam-distilled essential oils, rich in the oxygenated monoterpene 1,8-cineole, exhibited strong activity against Gram-negative bacteria [38]. Studies of edible flowers have highlighted their potential as sources of polyphenolic compounds with antioxidant and antimicrobial activities. Comparative evaluation of thirteen edible flowers from Japan demonstrated considerable variation in polyphenol content and antioxidant capacity, while several flower extracts also showed inhibitory effects against tested bacterial strains. The observed bioactivities were associated with the presence of phenolic and other bioactive constituents, supporting the potential of edible flowers as functional food ingredients with antioxidant and antimicrobial properties [54].

### 7.6. Anti-Inflammatory Activity

Neuroinflammation is increasingly recognized as an important contributor to depression and other neuropsychiatric disorders. *Hemerocallis citrina* exhibits promising anti-inflammatory effects, with increasing evidence implicating its flavonoid and phenylpropanoid constituents. In rats subjected to chronic unpredictable mild stress, phenylpropanoid- and flavonoid-enriched fractions of *H. citrina* significantly reduced the levels of the pro-inflammatory cytokines IL-6, IL-1β, and TNF-α in the hippocampus, together with decreased serum corticosterone, indicating attenuation of stress-associated neuroinflammation [8]. The isolated phenylpropanoids and flavonoids further demonstrated anti-neuroinflammatory activity, supporting these constituents as important contributors to the observed effects [8]. Independently, ethanol extracts of *H. citrina* attenuated the CUMS-induced elevation of IL-1β, IL-6, and TNF-α and suppressed indoleamine 2,3-dioxygenase activity in the frontal cortex and hippocampus [46]. More recently, Xu et al. isolated 12 constituents from *H. citrina* flower buds and demonstrated that several of these compounds significantly inhibited nitric oxide production in RAW264.7 macrophages, while some also exhibited DPPH radical-scavenging activity [55]. Collectively, these findings indicate that the flavonoid and phenolic constituents of *H. citrina* can modulate inflammatory responses through suppression of pro-inflammatory cytokines, excessive NO production, and IDO-related pathways, thereby potentially contributing to its neuroprotective and antidepressant effects.

### 7.7. Hepatoprotective Activity

Accumulating evidence indicates that *Hemerocallis citrina* flavonoids exert protective effects against hepatic fibrosis and oxidative injury. In a CCl_4_-induced liver fibrosis model, *H. citrina* total flavonoids significantly improved serum biochemical markers of liver function, including ALT, AST, GGT, and ALP, while enhancing SOD activity and reducing MDA levels. They also suppressed TGF-β1 expression and reduced hepatic PINP content, indicating attenuation of both oxidative stress and fibrogenic responses [56]. Complementary in vitro studies using H_2_O_2_-injured HL-7702 human liver cells showed that *H. citrina* total flavonoids improved cell viability, reduced MDA accumulation, and increased SOD activity. The treatment also downregulated LXRα and fatty acid synthase (FAS) expression, suggesting additional effects on oxidative damage and lipid metabolism [57]. Collectively, these findings support the hepatoprotective potential of *H. citrina* flavonoids through coordinated antioxidant, anti-fibrotic, and metabolic regulatory mechanisms.

### 7.8. Anti-Aging Activity

Oxidative stress is a major contributor to cellular senescence and age-related tissue damage, making the antioxidant properties of plant polyphenols relevant to potential anti-aging applications. *Hemerocallis citrina* is particularly rich in flavonoids and phenolic acids, and several studies have demonstrated strong antioxidant activity associated with these constituents. Phenolic-rich extracts obtained using ultrasonic-assisted 50% ethanol extraction exhibited the highest antioxidant capacity among the tested extraction approaches, with significant DPPH, FRAP, and ABTS radical-scavenging activities and the identification of 26 phenolic compounds, including eight phenolic acids and 18 flavonoids [37]. In addition, an ethyl acetate fraction of *H. citrina* containing abundant flavonoids and caffeic acid derivatives showed strong DPPH, ABTS, and reducing-power activities and protected cells against oxidative damage by reducing ROS accumulation [28]. More recently, several phenolic and flavonoid constituents isolated from *H. citrina*, including isoquercetin, isorhamnetin-3-O-β-D-galactoside, velutin, rutin, and neochlorogenic acid, demonstrated antioxidant activity in DPPH and ABTS radical-scavenging assays [58]. Collectively, these findings suggest that the flavonoid- and phenolic-rich fractions of *H. citrina* possess substantial antioxidant potential that may contribute to anti-aging and cellular-protective applications. However, direct evidence linking *H. citrina* flavonoids or phenolic compounds to specific anti-aging endpoints such as elastase inhibition, collagen synthesis, or cellular senescence remains limited and warrants further investigation.

### 7.9. Neuroprotective Activities

Phenolic acid derivatives and flavonoids from *Hemerocallis citrina* exhibit promising neuroprotective effects against chemically induced neuronal injury. In corticosterone- and glutamate-challenged PC12 cells, phenolic fractions significantly improved cell viability and reduced lactate dehydrogenase release in a dose-dependent manner. Phenolic acid derivatives and flavonoids showed comparable protective effects while differentially modulating neurotransmitter levels, with effects observed on dopamine, serotonin, noradrenaline, GABA, and acetylcholine [59]. More recently, the ethyl acetate fraction of the ethanol extract of *H. citrina* flower buds (HC-EA) demonstrated marked protection against amyloid-β (Aβ)-induced toxicity in *Caenorhabditis elegans*. HC-EA alleviated Aβ-induced paralysis, prolonged lifespan, reduced ROS accumulation, and enhanced glutathione peroxidase and proteasome activities. Mechanistically, these effects were associated with activation of the SKN-1 antioxidant pathway and enhanced proteasomal function, suggesting that *H. citrina* may protect against neurodegenerative stress through coordinated antioxidant and protein-quality-control mechanisms [60]. Collectively, these findings highlight the neuroprotective potential of *H. citrina* phenolics and flavonoids across cellular and whole-organism models.

### 7.10. Lactation-Promoting Activity

**Figure 5:**
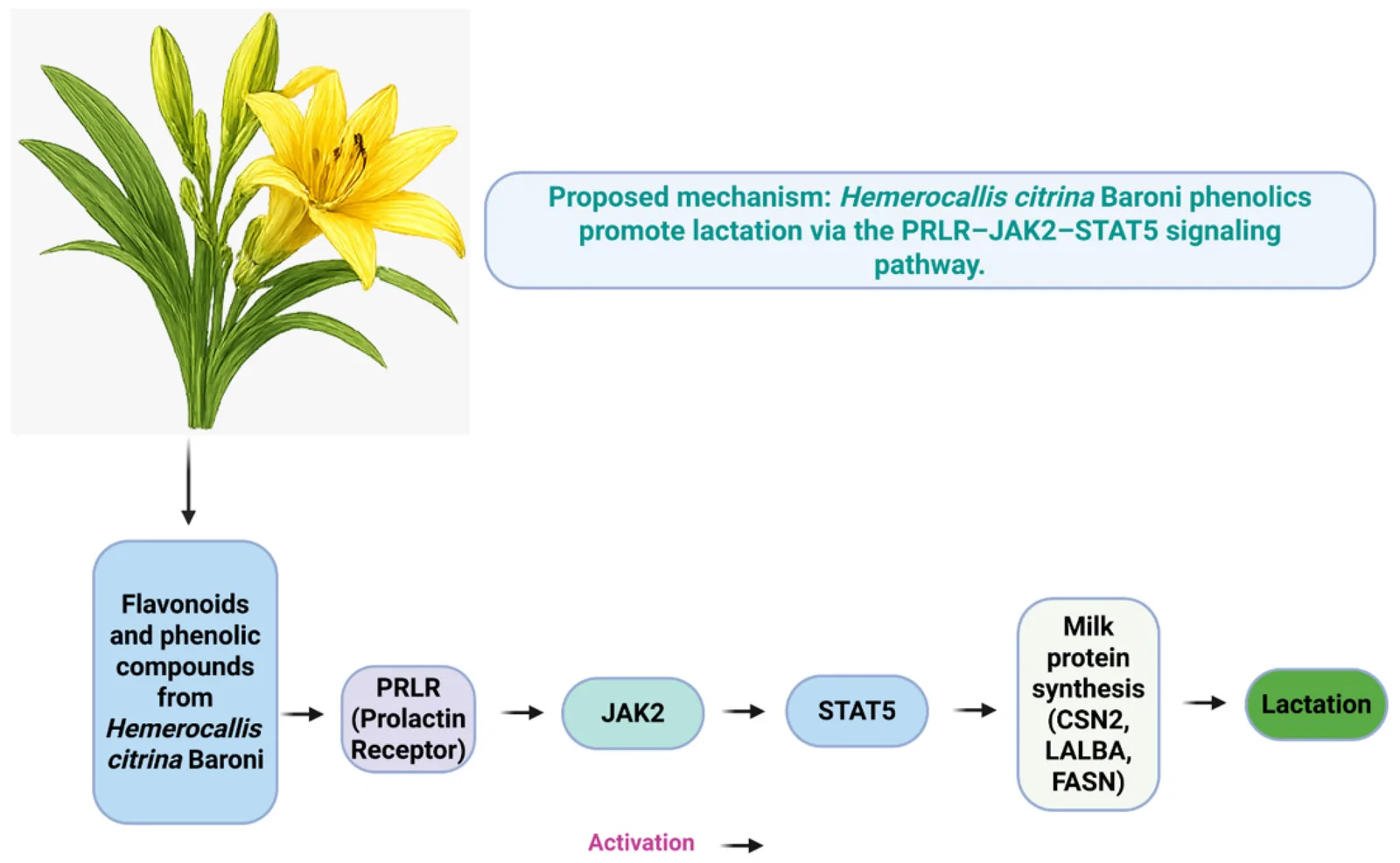
Proposed mechanism: *Hemerocallis citrina* Baroni phenolics promote lactation via the PRLR–JAK2–STAT5 signaling pathway.

Flavonoids and phenolic compounds from Hemerocallis citrina Baroni promote lactation via the PRLR–JAK2–STAT5 signaling pathway. Network pharmacology and experimental studies revealed that key flavonoids such as quercetin and kaempferol act through the prolactin signaling pathway, regulating core targets including JAK2, STAT3, and MAPK, thereby increasing serum prolactin (PRL) and oxytocin levels and improving milk production in lactation-deficient models [61]. Subsequent research further confirmed that flavonoids and phenols in daylily buds promote lactation via the PRLR/JAK2/STAT5 pathway, as shown in Figure 5 [62].

**Table 5:** Biological activities of polyphenols and flavonoids from *Hemerocallis citrina* Baroni.

Biological Activity	Active Extract/Compounds	Key Mechanisms/Effects	Experimental Model/Observation	Reference
**Antioxidant**	Ethanol/water extracts; supercritical CO_2_ extract; flavonoids & phenolic acids	↑ Glutathione in HUVE cells; scavenged DPPH, superoxide anion, hydroxyl radicals, H_2_O_2_; ↑ SOD & CAT; upregulation of Nrf2-related genes	HUVE cells; in vitro radical scavenging assays; BRL-3A cells	[27,30,35,37,41]
**Anti-tumor/Anticancer**	Water extract; methanol extract (roots)	Colchicine-mediated mitotic inhibition; induced cancer cell differentiation; inhibited S_180_ tumor proliferation; mitochondrial apoptosis (↑ Cytochrome C, BAX, Caspase-3/9; ↓ BCL-2); ↓ PKM2 & aerobic glycolysis; inhibition of Wnt/β-catenin pathway; G2/M arrest	Fibroblasts; breast/lung cancer cells; S_180_ tumor-bearing mice; HepG2 & Bel-7402 HCC cells	[40,41,42]
**Anti-depressant**	95%/90%/80%/75% ethanol extracts; total phenols (HCPE); phenylpropanoids & flavonoids; rutin + water extract	↓ Monoamine oxidase; ↑ 5-HT, NE, DA; ↑ BDNF-TrkB signaling; ↓ CORT & IDO; inhibition of NF-κB; modulation of gut microbiota (*Bacteroides* ↑, *Desulfovibrio* ↓); regulation of amino acid & energy metabolism	CORT-induced & CUMS rats; forced swim, sucrose preference, open-field tests	[8,23,36,39,45,46]
**Sleep improvement & Sedative**	75% ethanol extract; ethanol/water extract; root ethanol extract	Increased sleep duration; decreased locomotor activity via reduced catecholamine activity in cortex and brainstem	Mice; rats	[47,48,49,50,51]
**Antibacterial**	95% ethanol + chloroform extract (roots); 95%/75% ethanol extracts; steam-distilled essential oil	Inhibited *E. coli*, *P. aeruginosa*, *S. aureus*, *S. epidermidis*, *C. albicans*; anthraquinones, flavonoids, and oxygenated monoterpenes (1,8-cineole) as active constituents	In vitro antibacterial assays	[38,52,53,54]
**Anti-inflammatory**	75% ethanol ultrasonic extract; 95% ethanol extract	↓ Pro-inflammatory cytokines (IL-1β, IL-6, TNF-α) in frontal cortex; ↓ iNOS & COX-2; inhibited NO production in macrophages	CUMS rats; LPS-induced models; macrophages	[44,46,56]
**Hepatoprotective**	70% ethanol total flavonoid extract	↓ TGF-β1; ↑ SOD activity; ↓ LXRα and fatty acid synthase expression; reduced oxidative damage in liver cells	CCl_4_-induced liver fibrosis rats; H_2_O_2_-induced HL-7702 cells	[56,57]
**Anti-aging (anti-wrinkle)**	Ethanol (HF-EE) and water (HF-WE) extracts rich in polyphenols and flavonoids	Strong antioxidant activity (DPPH scavenging up to 87.20%); potent elastase inhibition (>88%); enhanced type I collagen synthesis; low cytotoxicity	Human dermal fibroblasts (CCD-986Sk); in vitro antioxidant assays	[58]
**Neuroprotection**	Phenolic acid derivatives and flavonoids from daylily flower extract, Ethyl acetate fraction of ethanol extract (HC-EA)	Dose-dependent neuroprotection against corticosterone- and glutamate-induced toxicity; phenolic acids elevated DA levels; flavonoids increased ACH and 5-HT levels; non-phenolic fractions showed no activity; ↓ Aβ transcription & protein levels; ↓ ROS; ↑ GPx & proteasome activity; upregulation of antioxidant (gst-4, skn-1) and proteasome genes; SKN-1 pathway activation	[PC12 cells], ↓ Aβ transcription & protein levels; ↓ ROS; ↑ GPx & proteasome activity; upregulation of antioxidant (gst-4, skn-1) and proteasome genes; SKN-1 pathway activation	[59,60]
**Lactation-promoting activity**	Flavonoids (quercetin, kaempferol) and phenolic compounds	Activation of prolactin signaling pathway (JAK2/STAT3/STAT5); ↑ serum PRL and oxytocin levels	CUMS-induced lactation deficiency in rats; bromocriptine-induced model	[61,62]

### 7.11. Major Flavonoid Components and Their Signaling Pathways in Metabolic-Associated Fatty Liver Disease: Mechanistic Insights from Hemerocallis citrina Baroni Flower Buds

**Figure 6:**
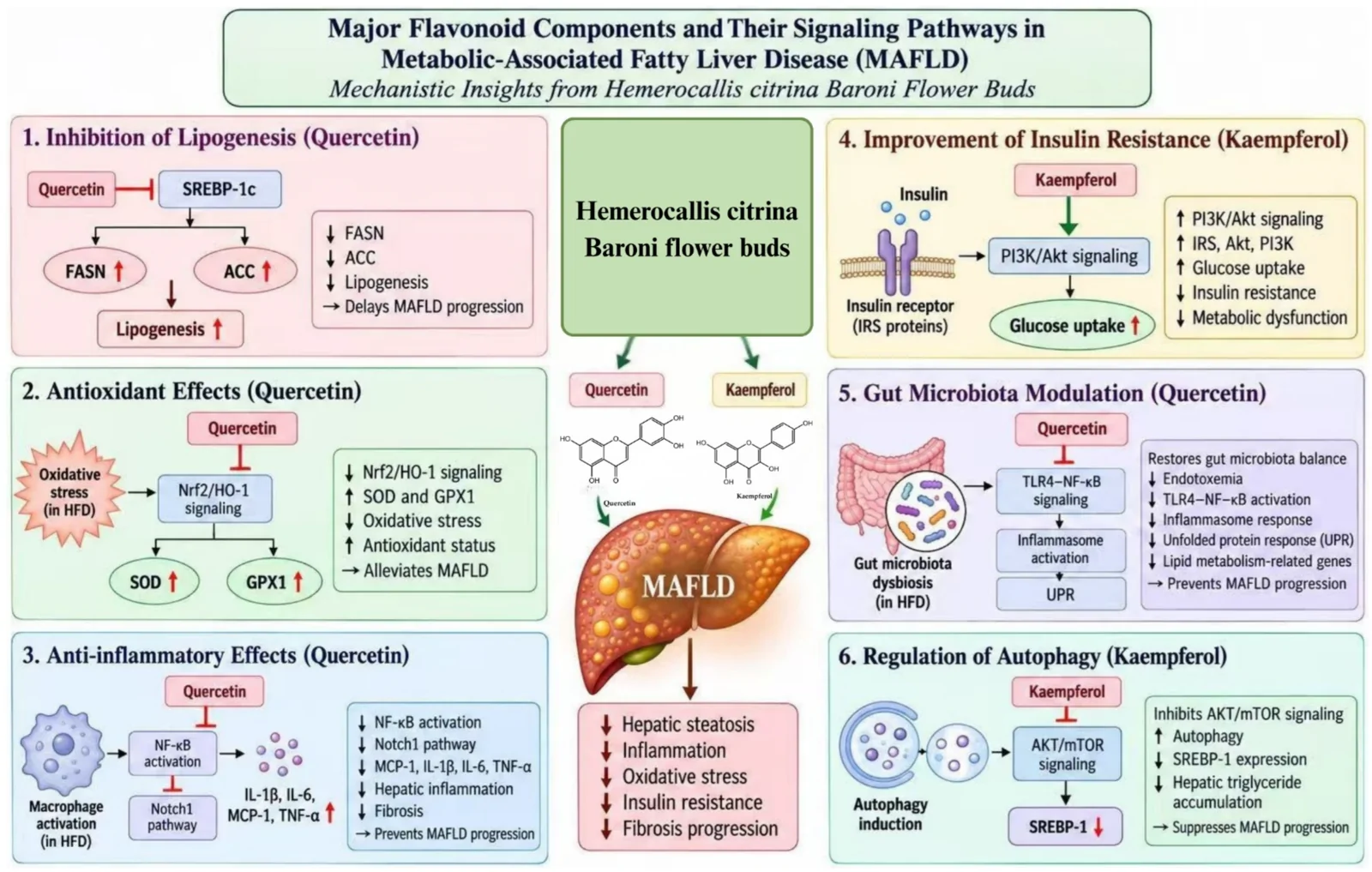
Major flavonoid components and their signaling pathways in metabolic-associated fatty liver disease (MAFLD) from *Hemerocallis citrina* Baroni flower buds.

The development of metabolic-associated fatty liver disease (MAFLD) is strongly linked to dysregulated lipid metabolism, oxidative stress, insulin resistance, chronic inflammation, and gut microbiota imbalance. As the most abundant flavonols in *Hemerocallis citrina* Baroni flower buds, quercetin and kaempferol have already been extensively reported to exert the same mechanistic actions in these diseases, as shown in Figure 6. These findings strongly support the potential of *H. citrina* flower buds as a rich natural source of these bioactive flavonoids, which can be directly applied to attenuate MAFLD progression.

As one of the most abundant flavonols in *Hemerocallis citrina* Baroni flower buds, quercetin has been shown to attenuate lipogenesis. In HepG2 cells overexpressing SREBP-1c, quercetin treatment (6 h) significantly reduced the levels of FASN and ACC proteins, thereby delaying the progression of MAFLD [63,64]. Quercetin, one of the most abundant flavonols in *Hemerocallis citrina* Baroni flower buds, exhibits strong antioxidant capacity and can significantly enhance the antioxidant status of mice fed a high-fat diet. It downregulates the Nrf2/HO-1 signaling pathway while upregulating the expression of superoxide dismutase (SOD) and glutathione peroxidase 1 (GPX1), thereby alleviating oxidative stress. In addition, quercetin regulates and upregulates PPARα and CPT-1A, promoting fatty acid β-oxidation and reducing lipid peroxidation-induced cellular damage [65]. Quercetin, as one of the most abundant flavonols in *Hemerocallis citrina* Baroni flower buds, effectively inhibits the abnormal expression of HFD-induced pro-inflammatory cytokines (IL-1β and IL-6). It suppresses the activation of the nuclear factor κB (NF-κB) signaling pathway and reduces macrophage-mediated liver inflammation and fibrosis by inhibiting the Notch1 pathway. Quercetin significantly decreases the expression of pro-inflammatory cytokines such as monocyte chemoattractant protein-1 (MCP-1), IL-1β, IL-6, and TNF-α, thereby alleviating hepatic inflammation and preventing the progression of MAFLD [66,67]. Kaempferol, one of the most abundant flavonols in *Hemerocallis citrina* Baroni flower buds, plays a key role in improving insulin resistance (IR). By activating the PI3K/Akt signaling pathway, kaempferol enhances insulin signaling through insulin receptor substrate (IRS) proteins and key mediators such as Akt and PI3K, thereby improving glucose uptake and alleviating metabolic dysfunction associated with IR [68]. Quercetin, one of the most abundant flavonols in *Hemerocallis citrina* Baroni flower buds, effectively regulates gut microbiota dysbiosis and alleviates MAFLD. It reverses the imbalance of gut microbiota and inhibits endotoxemia-mediated activation of the TLR4–NF-κB signaling pathway, thereby blocking the activation of the inflammasome response and the unfolded protein response (UPR). This leads to the downregulation of lipid metabolism-related gene expression and prevents the progression of MAFLD [69,70]. Kaempferol, one of the most abundant flavonols in *Hemerocallis citrina* Baroni flower buds, effectively regulates liver autophagy and suppresses MAFLD progression. As a potent inhibitor of the AKT/mTOR pathway, kaempferol induces autophagy in mouse liver cells by suppressing AKT/mTOR signaling and reduces hepatic triglyceride accumulation by downregulating SREBP-1 expression [71].

### 7.12. Anti-Depressant Mechanism of Total Phenol from Hemerocallis citrina Baroni Leaves (HLTP) via Modulation of the Microbiota–Gut–Brain Axis (MGBA) in Chronic Unpredictable Mild Stress (CUMS)-Induced Rats

Depression is one of the most prevalent psychiatric disorders worldwide and is closely associated with dysfunction of the microbiota–gut–brain axis (MGBA) [72]. The chronic unpredictable mild stress (CUMS) paradigm is widely recognized as a reliable preclinical model because it reproduces both behavioral and biochemical features of human depression [73]. Treatment with *Hemerocallis citrina* leaf total phenol (HLTP) significantly ameliorated depressive-like behaviors induced by chronic unpredictable mild stress (CUMS). These improvements included recovery of body weight and sucrose preference, shorter immobility in the forced swimming test (FST), reduced feeding latency in the novelty-suppressed feeding test (NSFT), and increased locomotor activity in the open field test (OFT) [74,75,76].

**Figure 7:**
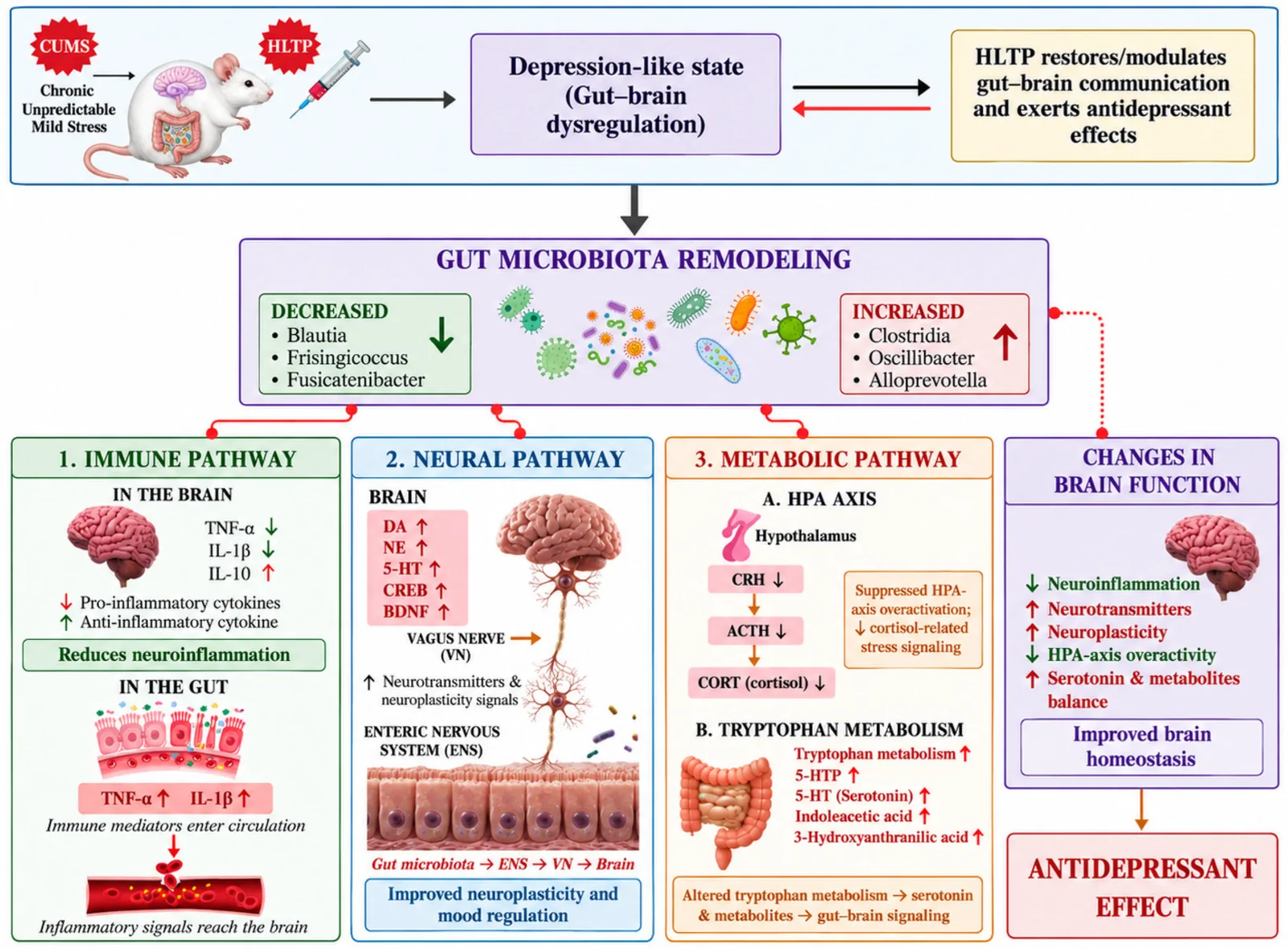
Anti-depressant mechanism of total phenol from *Hemerocallis citrina* Baroni leaves (HLTP) in chronic unpredictable mild stress (CUMS)-induced rats via modulation of the microbiota–gut–brain axis (MGBA). HLTP restores gut microbiota composition, enhances monoamine neurotransmitter levels, suppresses neuroinflammation, corrects HPA-axis hyperactivity, and normalizes tryptophan and nicotinate/nicotinamide metabolism, collectively alleviating depression-like behaviors.

As shown in Figure 7, HLTP exerts its antidepressant effects through three interconnected pathways: (1) immune pathway modulation, (2) neural pathway enhancement, and (3) metabolic pathway regulation. First, HLTP reversed gut microbiota dysbiosis by significantly increasing microbial diversity and richness while modulating the abundance of multiple taxa [77]. Notably, it restored the levels of beneficial genera such as *Alloprevotella* (positively correlated with 5-HT and BDNF) and *Monoglobus*, and reversed the enrichment of pathogenic genera such as *Blautia*, *Frisingicoccus*, and *Fusicatenibacter* [78,79,80]. Second, HLTP enhanced neural plasticity by increasing the levels of monoamine neurotransmitters (DA, NE, and 5-HT) and neurotrophic factors (BDNF and CREB) in the brain [81,82,83,84,85]. Third, HLTP suppressed hyperactivity of the hypothalamic–pituitary–adrenal (HPA) axis by reducing CRH, ACTH, and CORT levels [86,87], restored tryptophan metabolism (increased 5-HTP, serotonin, and metabolites), and modulated nicotinate/nicotinamide and porphyrin pathways [88,89,90,91,92]. Collectively, these findings demonstrate that HLTP exerts antidepressant effects in CUMS-induced rats primarily by modulating the MGBA, thereby improving neuroinflammation, restoring monoamine neurotransmitter balance, and correcting HPA-axis dysfunction.

## 8. Food and Industrial Applications

*Hemerocallis citrina* Baroni has an unusual dual identity as both an edible vegetable and a traditional medicinal plant. Its unopened flower buds, commonly known as Huanghuacai or daylily, have long been consumed in China as a fresh or dried vegetable and are also regarded as a functional food. The buds are traditionally boiled, steamed, stir-fried, incorporated into soups and stews, or dried to extend their shelf life [9,11]. This food–medicine continuum provides an important foundation for the modern development of *H. citrina* as a source of bioactive ingredients.

Recent metabolomic investigations further support the functional-food value of the edible flower buds. Jiang et al. identified 157 differentially accumulated metabolites during flower-bud development, including 49 flavonoids, 17 fatty acids, and 13 terpenoids. Notably, the abundance of several functional constituents changed substantially with developmental stage, demonstrating that harvest maturity is an important determinant of the nutritional and phytochemical quality of edible *H. citrina* buds [93]. The authors further suggested that the developmental dynamics of flavonoids and other metabolites should be considered when selecting raw material for functional-food production [93].

Postharvest processing represents another important factor governing the food quality of *H. citrina*. Conventional sun drying and hot-air drying facilitate long-term storage but can cause losses of thermolabile nutrients and bioactive compounds. A comparative study showed that freeze-drying better preserved polyphenols, flavonoids, carotenoids, and antioxidant capacity than hot-air or sun drying, whereas sun drying generated a richer volatile profile and therefore may offer advantages for flavor development [94]. These findings indicate that drying technology should be selected according to the desired balance between phytochemical retention, nutritional quality, antioxidant capacity, and sensory properties.

More recent processing research has focused on improving both drying efficiency and product safety. Vacuum-steam pulsed blanching shortened drying time, reduced enzymatic browning, promoted the release of phenolic compounds, and enhanced antioxidant activity compared with conventional steam blanching [95]. A 2026 study comparing different pretreatments before hot-air drying further demonstrated that blanching, color-preserving treatments, alkaline treatment, and ultrasound-assisted pretreatment could substantially reduce colchicine content while affecting color, polyphenol retention, microstructure, and drying behavior differently. The color-preserving treatment provided the best overall balance of safety, drying efficiency, color preservation, and polyphenol retention [96].

The chemical composition of *H. citrina* also provides opportunities for developing value-added functional foods and nutraceutical ingredients. Flavonoids and phenolic acids constitute major classes of secondary metabolites in the edible flower and are associated with antioxidant, anti-inflammatory, antidepressant, and other biological activities described above. Consequently, standardized extracts or phenolic-rich fractions may provide a basis for future functional beverages, dietary supplements, and other food-derived health products. However, such applications should be supported by appropriate safety evaluation, compositional standardization, bioavailability studies, and human clinical evidence before therapeutic claims are made. Traditional food use also provides a scientifically testable basis for some of the proposed health applications. In particular, *H. citrina* has traditionally been consumed by postpartum women to promote lactation. Fang et al. demonstrated that aqueous extracts of *H. citrina* promoted lactation in a rat model and identified 19 constituents in the active fractions; importantly, rutin and isoquercetin were individually shown to possess lactation-promoting activity. The proposed mechanism involved modulation of prolactin, progesterone, and prolactin-receptor expression [97,98]. These findings provide an example of how traditional dietary use can guide the identification of specific bioactive constituents, although clinical validation in humans remains necessary.

Overall, the current evidence supports *H. citrina* as a promising food and functional-food resource rather than merely a medicinal plant. Future industrial development should focus on optimized harvesting maturity, safe detoxification and drying processes, preservation of flavonoids and phenolic compounds, standardized extracts, and food-grade formulations that retain both nutritional and sensory quality.

## 9. Safety, Toxicity, Processing, and Species Authentication

### 9.1. Toxicological Concerns and Colchicine-Related Safety

Food safety is an important consideration in the utilization of *Hemerocallis citrina* Baroni, particularly because historical reports have associated the consumption of fresh flower buds with poisoning incidents. Colchicine has traditionally been proposed as the toxic constituent responsible for these incidents. However, subsequent analytical and genomic investigations have challenged this interpretation. Qing et al. reported that colchicine and its precursors were not detected in different tissues of *H. citrina* by high-resolution mass spectrometry, and no genes associated with the colchicine biosynthetic pathway were identified in the genome [31]. Similarly, Liu et al. comprehensively profiled metabolites from commercially available *H. citrina* flower buds using HPLC-Q-TOF-MS but did not report colchicine among the detected metabolites [34]. These findings indicate that the long-standing attribution of *H. citrina* poisoning specifically to colchicine should be interpreted cautiously and that the chemical basis of historical poisoning reports remains unresolved.

Despite this uncertainty, the reported occurrence of poisoning incidents emphasizes the importance of appropriate raw-material selection and processing. Processing studies have demonstrated that blanching and other pretreatments can substantially modify safety-related constituents as well as desirable quality characteristics of *H. citrina*. Vacuum-steam pulsed blanching has been reported to improve drying efficiency, reduce enzymatic browning, and influence the accumulation or release of plant chemical constituents [95]. More recently, hot-water blanching, steam blanching, color-preserving treatment, alkaline treatment, and ultrasound-assisted pretreatment were compared before hot-air drying, and all five pretreatments significantly reduced the measured colchicine content while producing different effects on drying behavior, color, microstructure, flavor, and polyphenol retention [96]. These findings support the importance of standardized processing conditions for the safe utilization of *H. citrina* as an edible plant.

Available toxicological studies provide additional, although predominantly preclinical, information regarding the safety of *H. citrina* extracts. Li et al. evaluated an ethanol extract of *H. citrina* in mice following repeated oral administration at 180, 360, and 720 mg/kg for up to 8 weeks. No significant abnormalities in body weight or relative organ weights were observed, and the study provided no major evidence of liver or kidney toxicity under the experimental conditions [99]. In another study, a 75% ethanol extract of *H. citrina* was evaluated for acute toxicity, and no mortality was observed following oral administration of up to 5000 mg/kg. Histopathological examination after administration of 5000 mg/kg for either 1 day or 21 consecutive days did not reveal significant lesions in the liver, kidney, or spleen [36]. These findings provide useful evidence regarding the tolerability of specific *H. citrina* extracts in experimental animals; however, they should not be interpreted as establishing a safe human dose or as demonstrating that all *H. citrina* preparations are safe.

Other safety considerations may arise from differences in plant material, developmental stage, geographical origin, extraction conditions, and processing history, all of which can influence the chemical composition of *H. citrina*. Therefore, the presence of beneficial flavonoids and phenolic compounds should not be interpreted as evidence of unrestricted safety. For food and nutraceutical applications, appropriate botanical authentication, control of raw materials, standardized processing, chemical characterization, and toxicological evaluation are necessary. At present, human clinical safety data and well-established human intake limits for *H. citrina* preparations remain limited. Accordingly, specific safe doses should not be proposed in the absence of sufficient human evidence.

### 9.2. Processing-Related Safety Considerations

Processing is particularly important because *H. citrina* flower buds are commonly consumed after cooking, drying, or other forms of treatment. Recent research has demonstrated that pretreatment before hot-air drying can simultaneously affect safety-related constituents, drying characteristics, color, flavor, and retention of bioactive compounds [96]. Vacuum-steam pulsed blanching has also been shown to improve drying efficiency and reduce enzymatic browning while affecting the accumulation and release of chemical constituents [95]. These findings indicate that processing should be optimized as a balance between safety, phytochemical preservation, sensory quality, and storage stability rather than solely maximizing the retention of bioactive constituents.

The conflicting evidence concerning colchicine further illustrates the need for validated analytical methods and appropriate reference standards in safety assessment. Earlier reports attributed poisoning associated with fresh *H. citrina* flower buds to colchicine, whereas subsequent high-resolution mass-spectrometric and genomic investigations did not support the presence of colchicine or its biosynthetic pathway in the species [31,34]. Consequently, future studies should employ orthogonal analytical approaches and authenticated reference standards to establish the identity and concentration of any suspected toxic constituents.

### 9.3. Species Authentication and Differentiation from Other Hemerocallis Species

Species authentication is an important component of both quality control and food safety because closely related *Hemerocallis* species can be difficult to distinguish after drying or processing. In particular, *H. citrina* and *Hemerocallis fulva* (L.) L. may be confused in processed materials because diagnostic morphological characteristics can be lost. Chemical and molecular approaches can therefore complement conventional morphological identification. Metabolomic profiling, chromatographic fingerprints, DNA-based methods, and other molecular approaches have been investigated for distinguishing *H. citrina* from related *Hemerocallis* species [12,34]. Recent research on *H. citrina* polysaccharides has further highlighted the potential value of polysaccharide composition, structural characteristics, and molecular-weight distribution as complementary authentication markers [15].

Regional chemical variation may also provide useful information for quality assessment. Metabolomic studies have demonstrated differences in the chemical profiles of *H. citrina* collected from different production areas, indicating that chemical fingerprints may contribute to the assessment of geographical origin and raw-material quality. Such approaches may be particularly valuable when morphological identification is difficult after drying or processing. Therefore, validated combinations of morphological, chromatographic, metabolomic, DNA-based, and chemical markers may provide a more reliable authentication framework for dried flower buds, extracts, functional foods, and nutraceutical products.

### 9.4. Overall Safety Evidence and Research Needs

Overall, the available evidence indicates that the safety profile of *H. citrina* should be evaluated independently from the beneficial biological activities reported for its flavonoids and phenolic compounds. Historical poisoning reports, the unresolved identity of the constituents responsible for such incidents, processing-dependent changes in chemical composition, differences among plant materials, and the limited availability of human safety data all warrant continued investigation. Current toxicological evidence is mainly derived from animal studies of specific extracts, whereas systematic human safety evaluations, standardized exposure assessments, and long-term safety studies remain insufficient. Future research should therefore prioritize the identification of the constituents responsible for historical poisoning reports, validation of analytical methods for suspected toxic compounds, characterization of processing-dependent changes, establishment of standardized safety and quality markers, and well-designed human safety studies. Such evidence will be essential for the responsible development of *H. citrina* as a food, functional-food ingredient, or nutraceutical resource.

## 10. Conclusions

In conclusion, this review provides a comprehensive and up-to-date synthesis of flavonoids and phenolic compounds reported from *Hemerocallis citrina* Baroni, covering their phytochemical diversity, extraction and analytical characterization, biological activities, safety considerations, and potential food and health applications from 2000 to 2026. Advances in chromatographic, mass spectrometric, and metabolomic technologies have revealed a diverse range of polyphenolic constituents, including quercetin, kaempferol, isorhamnetin derivatives, rutin, hesperidin, and caffeoyl- and *p*-coumaroylquinic acid derivatives. These compounds and polyphenol-rich extracts have been associated with antioxidant, anti-inflammatory, antidepressant, sleep-related, hepatoprotective, and neuroprotective effects in experimental studies.

However, the current evidence should be interpreted primarily within a preclinical context. Most reported biological activities of *H. citrina* and its flavonoid- and phenolic-rich extracts have been investigated using in vitro assays, cultured cells, animal models, or other non-human experimental systems. For example, reported antidepressant effects have been demonstrated in animal behavioral models and supported by mechanistic investigations, rather than by controlled human clinical trials. Although these findings provide important evidence for biological potential, they cannot by themselves establish clinical efficacy in humans.

Human evidence concerning the efficacy, pharmacokinetics, bioavailability, metabolism, dose–response relationships, long-term safety, and therapeutic effectiveness of *H. citrina* flavonoids and phenolic compounds remains limited. In addition, differences in plant material, cultivar, geographical origin, extraction procedures, processing conditions, compound identification, and chemical standardization may contribute to variability among reported findings. The translation of promising experimental results into functional foods, nutraceuticals, or therapeutic applications therefore requires further validation.

Future research should prioritize standardized and chemically characterized extracts, validated quantitative analytical methods, investigation of absorption and metabolism, food-grade delivery systems, processing optimization, and systematic toxicological evaluation. Importantly, well-designed human studies, including appropriately controlled clinical trials, are needed to determine whether the biological effects observed in experimental models can be reproduced in humans and to establish clinically relevant dose ranges, efficacy, bioavailability, and long-term safety. Overall, *H. citrina* represents a promising source of flavonoids and phenolic compounds for food and health-related applications, but its clinical potential should be considered preliminary until supported by robust human evidence.

## Figures and Tables

**Table 1:** Nutrient composition of *Hemerocallis citrina* (per 100 g edible portion) [15].

Nutritional Type	Project	Content	Ranking Among Similar Plants	Mean of the Same Category
**Energy and related components**	Edible	98%	17	85%
	Water	40.30 g	60	86.8 g
	Energy	975 kJ	4	204 kJ
	Protein	19.4 g	2	3.2 g
	Fat	1.4 g	4	0.4 g
	Ash	4.0 g	3	1.3 g
	Carbohydrate	34.9 g	5	8.3 g
**Vitamins**	Carotene	1840.0 µg	11	1061.6 µg
	Thiamin	0.05 mg	15	0.04 mg
	Riboflavin	0.21 mg	5	0.09 mg
	Niacin	3.10 mg	1	0.62 mg
	Vitamin C	10.0 mg	44	25.0 mg
**Mineral**	Ca	301 mg	2	84 mg
	P	216 mg	3	56 mg
	K	610 mg	2	254 mg
	Na	59.2 mg	24	66.0 mg
	Mg	85 mg	3	32 mg
	Fe	8.10 mg	3	2.4 mg
	Zn	3.99 mg	2	0.74 mg
	Se	4.02 µg	3	1.07 µg
	Cu	0.37 mg	5	0.17 mg
	Mn	1.21 mg	5	0.54 mg
Source: https://nlc.chinanutri.cn.				

**Table 2:** Individual flavonoids and phenolic/polyphenolic compounds reported in *Hemerocallis citrina* Baroni.

No.	Compound Name	Structural Class	Plant Part	Extraction/Isolation	Identification Method	Reference
1	Hemeroquinone A	Novel anthraquinone derivative	Flower buds	Phytochemical isolation	HR-ESI-MS, NMR, ECD	[26]
2	Hemeroquinone B	Novel anthraquinone derivative	Flower buds	Phytochemical isolation	HR-ESI-MS, NMR, ECD	[26]
3	Hemerophenan A	Novel phenanthrene derivative	Flower buds	Phytochemical isolation	HR-ESI-MS, NMR, ECD	[26]
4	2,4-Dihydroxy-6-(4-hydroxyphenethyl)-methyl benzoate	Phenolic acid derivative	Flower buds	Phytochemical isolation	Spectroscopic analysis	[26]
5	3-Methyl-2,4-dihydroxy-6-(4-hydroxyphenethyl)-methyl benzoate	Phenolic acid derivative	Flower buds	Phytochemical isolation	Spectroscopic analysis	[26]
6	(+)-Pinoresinol	Furofuran lignan	Flower buds	Phytochemical isolation	Spectroscopic analysis	[26]
7	(+)-Epipinoresinol	Furofuran lignan	Flower buds	Phytochemical isolation	Spectroscopic analysis	[26]
8	Pinoresinol-4-O-β-D-glucoside	Lignan glycoside	Flower buds	Phytochemical isolation	Spectroscopic analysis	[26]
9	Cinnamic acid	Hydroxycinnamic acid	Flower buds	Phytochemical isolation	Spectroscopic analysis	[26]
10	Hemerodiphenylethane A	Novel diphenylethane	Stems and leaves	Phytochemical isolation	HR-ESI-MS, 1D/2D-NMR, ECD	[27]
11	Hemerodiphenylethane B	Novel diphenylethane	Stems and leaves	Phytochemical isolation	HR-ESI-MS, 1D/2D-NMR, ECD	[27]
12	Hemerodiphenylethane C	Novel diphenylethane	Stems and leaves	Phytochemical isolation	HR-ESI-MS, 1D/2D-NMR, ECD	[27]
13	Hemerodiphenylethane D	Novel diphenylethane	Stems and leaves	Phytochemical isolation	HR-ESI-MS, 1D/2D-NMR, ECD	[27]
14	5-O-Caffeoylquinic acid (Chlorogenic acid)	Caffeoylquinic acid	Dried flowers (ethyl acetate extract)	70% ethanol ultrasonication → ethyl acetate fraction	UPLC-PDA/MS	[28]
15	Gallic acid	Hydroxybenzoic acid	Dried flowers (ethyl acetate extract)	70% ethanol ultrasonication → ethyl acetate fraction	UPLC-PDA/MS	[28]
16	4-O-p-Coumaroylquinic acid	p-Coumaroylquinic acid	Dried flowers	70% ethanol ultrasonication → ethyl acetate fraction	UPLC-PDA/MS	[28]
17	1-Cyclohexene-1-carboxylic acid	Unsaturated carboxylic acid	Dried flowers	70% ethanol ultrasonication → ethyl acetate fraction	UPLC-PDA/MS	[28]
18	Quercetin 3,7-di-O-β-D-glucopyranoside	Flavonol diglycoside	Dried flowers	70% ethanol ultrasonication → ethyl acetate fraction	UPLC-PDA/MS	[28]
19	Phloretin 2′-O-β-D-xylopyranosyl-(1→6)-β-D-glucopyranoside	Dihydrochalcone diglycoside	Dried flowers	70% ethanol ultrasonication → ethyl acetate fraction	UPLC-PDA/MS	[28]
20	Kaempferol-3-O-galactoside	Flavonol glycoside	Dried flowers	70% ethanol ultrasonication → ethyl acetate fraction	UPLC-PDA/MS	[28]
21	Kaempferol 3-O-rutinoside	Flavonol diglycoside	Dried flowers	70% ethanol ultrasonication → ethyl acetate fraction	UPLC-PDA/MS	[28]
22	Astragalin (Kaempferol-3-O-β-D-glucopyranoside)	Flavonol glycoside	Dried flowers	70% ethanol ultrasonication → ethyl acetate fraction	UPLC-PDA/MS	[28]
23	Isorhamnetin 3-O-glucoside	Flavonol glycoside	Dried flowers	70% ethanol ultrasonication → ethyl acetate fraction	UPLC-PDA/MS	[28]
24	Quercetin-3-O-α-L-arabinopyranoside	Flavonol glycoside	Flowers from different areas	50% methanol ultrasonication (30 min, RT)	UHPLC-QQQ-MS/MS	[29]
25	Kaempferol-3-O-β-D-galactopyranoside	Flavonol glycoside	Flowers from different areas	50% methanol ultrasonication (30 min, RT)	UHPLC-QQQ-MS/MS	[29]
26	Kaempferol-3-O-β-D-glucopyranoside	Flavonol glycoside	Flowers from different areas	50% methanol ultrasonication (30 min, RT)	UHPLC-QQQ-MS/MS	[29]
27	Isorhamnetin-3-O-β-D-galactopyranoside	Flavonol glycoside	Flowers from different areas	50% methanol ultrasonication (30 min, RT)	UHPLC-QQQ-MS/MS	[29]
28	Isorhamnetin-3-O-β-D-glucopyranoside	Flavonol glycoside	Flowers from different areas	50% methanol ultrasonication (30 min, RT)	UHPLC-QQQ-MS/MS	[29]
29	Kaempferol-3-O-[α-L-rhamnopyranosyl-(1→6)]-[α-L-rhamnopyranosyl-(1→2)]-β-D-glucopyranoside	Flavonol triglycoside	Flowers from different areas	50% methanol ultrasonication (30 min, RT)	UHPLC-QQQ-MS/MS	[29]
30	Isorhamnetin-3-O-[α-L-rhamnopyranosyl-(1→6)]-β-D-glucopyranoside	Flavonol diglycoside	Flowers from different areas	50% methanol ultrasonication (30 min, RT)	UHPLC-QQQ-MS/MS	[29]
31	Quercetin-3-O-β-D-galactopyranoside (Hyperoside)	Flavonol glycoside	Flowers from different areas	50% methanol ultrasonication (30 min, RT)	UHPLC-QQQ-MS/MS	[29]
32	Quercetin-3-O-β-D-glucopyranoside (Isoquercitrin)	Flavonol glycoside	Flowers from different areas	50% methanol ultrasonication (30 min, RT)	UHPLC-QQQ-MS/MS	[29]
33	Crypto-chlorogenic acid (4-O-Caffeoylquinic acid)	Caffeoylquinic acid	Flowers from different areas	50% methanol ultrasonication (30 min, RT)	UHPLC-QQQ-MS/MS	[29]
34	3-O-(Z)-p-Coumaroylquinic acid	p-Coumaroylquinic acid	Flowers from different areas	50% methanol ultrasonication (30 min, RT)	UHPLC-QQQ-MS/MS	[29]
35	3-O-(E)-p-Coumaroylquinic acid	p-Coumaroylquinic acid	Flowers from different areas	50% methanol ultrasonication (30 min, RT)	UHPLC-QQQ-MS/MS	[29]
36	4-O-(E)-p-Coumaroylquinic acid	p-Coumaroylquinic acid	Flowers from different areas	50% methanol ultrasonication (30 min, RT)	UHPLC-QQQ-MS/MS	[29]
37	4-O-(Z)-p-Coumaroylquinic acid	p-Coumaroylquinic acid	Flowers from different areas	50% methanol ultrasonication (30 min, RT)	UHPLC-QQQ-MS/MS	[29]
38	5-O-(E)-p-Coumaroylquinic acid	p-Coumaroylquinic acid	Flowers from different areas	50% methanol ultrasonication (30 min, RT)	UHPLC-QQQ-MS/MS	[29]
39	4-Hydroxycinnamic acid	Hydroxycinnamic acid	Flowers	Methanol ultrasonication	UPLC-Q-TOF-MS/MS	[29,33]

**Table 3:** Aggregate phytochemical profiling and genomic evidence for *Hemerocallis citrina* Baroni.

No.	Finding	Evidence Category	Plant Material/Context	Method/Evidence	Reference
1	6 phenolic acids and 71 flavonoids	Aggregate phytochemical profiling	Flower material	Comprehensive phytochemical profiling; molecular docking analysis	[30]
2	20 genes potentially involved in rutin biosynthesis	Genomic/biosynthetic evidence	Whole-plant genome	Chromosome-level genome assembly (3.77 Gb); integrated genomic and metabolomic analysis	[31]
3	44 flavonoid compounds, including rutin, kaempferol-, quercetin-, and related derivatives	Aggregate flavonoid profiling	Commercially available flower buds	Multi-strategy screening using non-targeted, accurate-targeted, and extensive-targeted approaches; HPLC-Q-TOF-MS	[34]

**Table 4:** Extraction procedures for flavonoids and polyphenols from *Hemerocallis citrina* Baroni.

Target Compounds/Extract Type	Plant Material	Extraction Solvent & Conditions	Additional Processing/Fractionation	Analytical Method Used	Reference
Polyphenols, flavonoids and phenylpropanoids, including isolated compounds	Flower buds/flowers	Solvent extraction; repeated extraction; solvent partitioning	Column chromatography and purification	HR-ESI-MS, 1D/2D-NMR, ECD; UHPLC-Q-TOF-MS/MS	[26,29]
Phenolic acids and flavonoids, including hyperoside, isoquercitrin, astragalin, rutin, gallic acid and chlorogenic acid	Powdered dried flowers	70% ethanol; ultrasonication, 40 min, 55 °C; 3 times	Concentration; sequential partitioning with *n*-hexane, ethyl acetate and *n*-butanol	UPLC-PDA/MS	[28]
Flavonoids and phenolic acids	Flowers from different geographical areas	50% methanol; ultrasonication, 30 min, room temperature	Direct analysis after extraction	UHPLC-Q-TOF-MS/MS and UHPLC-QQQ-MS/MS	[29]
Phenolic acids and flavonoids	*H. citrina* flowers	Phenolic extraction using soybean-protein-isolate-based system	Extract incorporated into SPI-based emulsion	Chromatographic/MS-based phenolic profiling	[30]
Phenolic acids, including 4-hydroxycinnamic acid	Dry flower buds from Sichuan, China	Methanol; ultrasonication, 40 kHz, 30 min	Direct analysis; NT, AT and ET screening	UPLC-Q-TOF-MS/MS	[29,33]
Flavonols and other polyphenols	Commercially available flower buds	Methanol–water extraction; ultrasonication, 60 min	Direct analysis; NT, AT and ET screening	HPLC-Q-TOF-MS	[34]
Phenylpropanoids, particularly 3-O-*p*-coumaroylquinic acid and 4-O-*p*-coumaroylquinic acid	*H. citrina* material	Water extraction	Fractionation to obtain 35% ethanol-eluted fraction	Chemical characterization of phenylpropanoids	[35]
Flavonoid-rich extract	*H. citrina* flowers	Hydroalcoholic extraction using different ethanol concentrations	Comparative preparation of extracts	HPLC	[36]
Phenolic acids and flavonoids	*H. citrina* flowers	50% ethanol or water; ultrasound-assisted extraction; enzyme-assisted extraction	Comparison of four extraction approaches	LC-QTOF-MS/MS	[37,38]
Phenylpropanoid- and flavonoid-enriched fractions	Air-dried flower buds	80% ethanol; cold-dipping, 3 × 48 h	AB-8 macroporous resin chromatography	UHPLC-Q-TOF-MS; HPLC-DAD; NMR; HR-ESI-MS	[8]

## Data Availability

No new data were created or analyzed in this study. Data sharing is not applicable to this article.
